# Treatment sequences for advanced renal cell carcinoma: A health economic assessment

**DOI:** 10.1371/journal.pone.0215761

**Published:** 2019-08-29

**Authors:** Baris Deniz, Apoorva Ambavane, Shuo Yang, Arman Altincatal, Justin Doan, Sumati Rao, M. Dror Michaelson

**Affiliations:** 1 Evidera, Inc., Bethesda, Maryland, United States of America; 2 Evidera, Inc., London, England, United Kingdom; 3 Bristol-Myers Squibb, Princeton, New Jersey, United States of America; 4 Massachusetts General Hospital Cancer Center, Hematology/Oncology, Boston, Massachusetts, United States of America; University of Washington, UNITED STATES

## Abstract

**Objective:**

Advanced renal cell carcinoma (RCC) is commonly treated with vascular endothelial growth factor or mammalian target of rapamycin inhibitors. As new therapies emerge, interest grows in gaining a deeper understanding of treatment sequences. Recently, we developed a patient-level, discretely integrated condition event (DICE) simulation to estimate survival and lifetime costs for various cancer therapies, using a US payer perspective. Using this model, we explored the impact of treatments such as nivolumab and cabozantinib, and compared the clinical outcomes and cost consequences of commonly used treatment algorithms for patients with advanced RCC.

**Methods:**

Included treatment sequences were pazopanib or sunitinib as first-line treatment, followed by nivolumab, cabozantinib, axitinib, pazopanib or everolimus. Efficacy inputs were derived from the CheckMate 025 trial and a network meta-analysis based on available literature. Safety and cost data were obtained from publicly available sources or literature.

**Results:**

Based on our analysis, the average cost per life-year (LY) was lowest for sequences including nivolumab (sunitinib → nivolumab, $75,268/LY; pazopanib → nivolumab, $84,459/LY) versus axitinib, pazopanib, everolimus and cabozantinib as second-line treatments. Incremental costs per LY gained were $49,592, $73,927 and $30,534 for nivolumab versus axitinib, pazopanib and everolimus-containing sequences, respectively. The model suggests that nivolumab offers marginally higher life expectancy at a lower cost versus cabozantinib-including sequences.

**Conclusion:**

Treatment sequences using nivolumab in the second-line setting are less costly compared with sequential use of targeted agents. In addition to efficacy and safety data, cost considerations may be taken into account when considering treatment algorithms for patients with advanced RCC.

## Introduction

Globally, kidney cancer is responsible for 2.4% of all adult malignancies, with approximately 338,000 new cases and 114,000 deaths annually [1]. Renal cell carcinoma (RCC) is the most common type of kidney cancer [2], with a poor prognosis: the 5-year relative survival rate is ~12% for metastatic RCC [3]. Historically, in the pre-targeted therapy era, median survival for people with metastatic RCC was ~8 months with no treatment [4] or ~13 months with immunotherapy [5].

Treatment of advanced RCC has evolved with the development of targeted therapies including vascular endothelial growth factor receptor tyrosine kinase inhibitors (TKIs), such as bevacizumab, sunitinib, sorafenib, pazopanib, axitinib, cabozantinib and lenvatinib or mammalian target of rapamycin inhibitors, such as everolimus and temsirolimus [6].

Although targeted agents have significantly improved progression-free survival (PFS) and overall survival (OS), there remains unmet need for patients who have not responded to previous targeted therapy [6]. In the setting of disease progression, cabozantinib is an option for patients who progress rapidly. In a phase III clinical trial, cabozantinib improved PFS (hazard ratio [HR] 0.58; 95% confidence interval [CI], 0.45–0.75; *P*<0 .001) and OS (HR 0.67; 95% CI, 0.51–0.89; *P* = 0.005) compared with everolimus [7,8]. An addition to the second-line therapeutic armamentarium is nivolumab, an immune checkpoint inhibitor (anti-PD-1), which was approved in the United States for use in pretreated advanced RCC [9]. In the phase III CheckMate 025 trial, nivolumab demonstrated superior OS compared with everolimus (HR 0.73; 98.5% CI, 0.57–0.93; *P* = 0.002) [10].

Despite the availability of multiple classes of treatment options, there is paucity of data on sequencing strategies for patients who have progressed on initial targeted therapy. In addition to health burden, RCC is associated with significant economic burden. In the United States, the annual economic burden of all RCC is estimated at $0.60 billion to $5.19 billion, with per-patient costs of $16,488 to $43,805 [11]. As new, more effective targeted treatment options become available, patients will likely continue to be managed with multiple treatment lines. Therefore, it will be essential to understand which treatment sequences are optimal with respect to costs and health outcomes.

A limited number of studies have evaluated treatment sequencing, and no published analyses have evaluated the economic burden of introducing nivolumab within the context of treatment sequences. Our objective was to develop a health economic model evaluating the cost and health outcomes associated with commonly used treatment sequences for patients with advanced RCC.

## Methods

### Model overview

**Fig 1:**
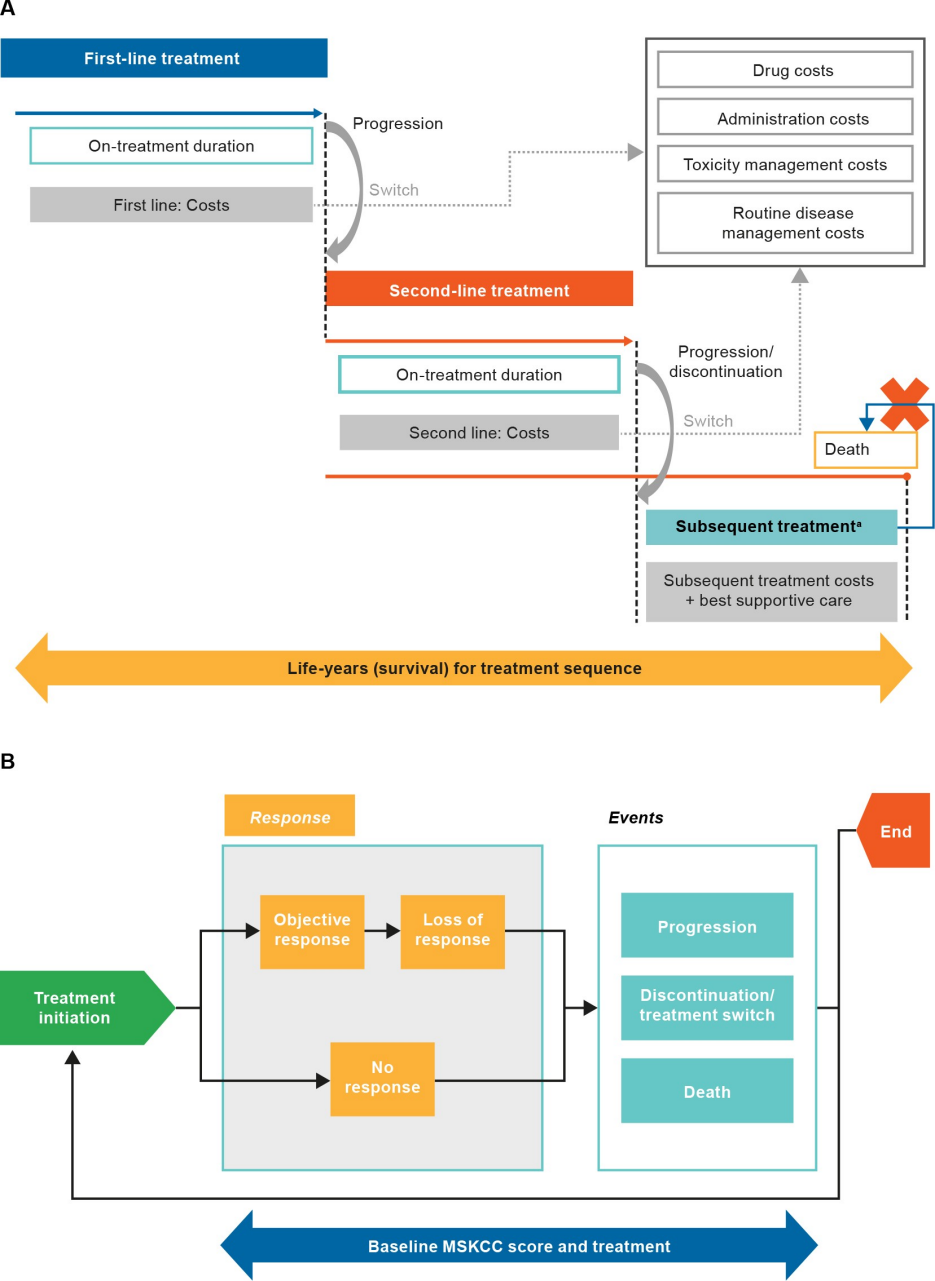
**Model flow (A) and structure during first- and second-line treatment (B).** ^a^A mix of rescue therapy patients may receive treatment post second line. This is assumed to have no effect on survival.

A health economic model was developed to estimate costs and health outcomes associated with various treatment sequences for advanced RCC during a patient’s lifetime (i.e., time horizon of 25 years; discounted at 3.0% [12] per annum) (Fig 1A).

The model adopts a US third-party payer perspective, with all costs provided in 2017 US dollars. The analysis population comprised patients with advanced RCC who were treatment-naïve at the start of the model and received pazopanib or sunitinib, two commonly used TKIs in frontline setting, as their first line treatment. The model considers treatment patterns following first-line pazopanib or sunitinib use reflecting current clinical practice, and is aligned with National Comprehensive Cancer Network (NCCN) clinical guidelines [13]. The most commonly used treatment patterns included in this study were determined via analysis of real-world databases, registries (Surveillance, Epidemiology, and End Results [SEER] program and MarketScan) (Bristol-Myers Squibb, data on file), and clinical opinion. The model includes treatment sequences composed of two lines of active treatment: sunitinib or pazopanib as first-line treatment, followed by everolimus, pazopanib, axitinib, nivolumab or cabozantinib as second-line options.

### Model structure

Studies have shown that advanced RCC prognoses depend on factors such as patient characteristics and disease severity [2,3,14]. Treatment guidelines, including NCCN, recommend considering prognostic indicators (e.g., Memorial Sloan Kettering Cancer Center [MSKCC] risk score or the International Metastatic Renal Cell Carcinoma Database Consortium score) in treatment decisions [13]. Furthermore, in other indications where immune-oncology therapies are used, studies suggest that tumor response (level of response and its durability) may be correlated with progression and survival [15,16].

To capture the complex underlying prognostic dynamics of advanced RCC, a patient-level, discrete event simulation was developed using the discretely integrated condition event (DICE) platform [17]. DICE simulation can capture the heterogeneity in patient populations and correlations between disease milestones in a flexible and intuitive manner, as it integrates “conditions” that persist over time (e.g., response and MSKCC status) and “events” associated with management and disease course (e.g., response achievement, treatment discontinuation and death) [17]. The DICE method provides the structural flexibility that enables integration of key disease milestones that are time-dependent (e.g., time to response, duration of response), and patient characteristics (e.g., MSKCC score at baseline). Furthermore, with DICE, patient risk profiles can be updated based on the individual disease and treatment experience in a computationally efficient way, without introducing unrealistic structural assumptions.

The model starts by simulating 1000 patients to whom various risk scores (based on CheckMate 025 distribution of MSKCC risk score [10]–Supplementary Material A in S1 Appendix) are assigned, followed by their first-line treatment. The progression (proxy for treatment discontinuation) and survival times are estimated based on efficacy of the corresponding treatment. When progression occurs, patients switch to second-line treatment, and time to progression (TTP) and survival times are updated based on new efficacy. While on treatment, patients may experience adverse events (AEs) based on treatment they are receiving. During the course of the model, costs related to treatment, administration, toxicity management, and disease management (based on progression status) are estimated for each patient. Treatment duration is determined by estimating time to treatment discontinuation (TTD) (for nivolumab and everolimus) or by using time of progression as a proxy for discontinuation (for all other therapies).

Patients who progress on second-line treatment receive subsequent treatment composed of a mix of salvage therapies. The subsequent treatment profile was obtained from CheckMate 025 trial data based on therapies provided to patients following discontinuation of nivolumab or everolimus. In the model, it was assumed that survival estimates were not impacted by salvage therapies, and associated treatment costs were accounted for within the overall management costs.

At the start of second-line treatment with nivolumab or everolimus, the model estimates whether patients achieve objective response (partial response or higher as per Response Evaluation Criteria in Solid Tumors [RECIST] v1.1 [10]–Supplementary Material B in S1 Appendix). For patients with objective response, the time to achieve response and subsequently, time to loss of response, are estimated. The model also estimates TTP, TTD, and time to death based on response status, duration of response, and the patient’s baseline MSKCC risk score (Fig 1B). For all other second-line therapies considered in the model, TTP and OS are estimated solely based on the assigned treatment, since relationships between response level and disease milestones are not available in the published literature. Supplementary Material C in S1 Appendix presents the detailed model path diagram.

### Statistical analysis: Efficacy inputs

Efficacy inputs for first-line treatments were based on the COMPARZ trial, a head-to-head comparison of sunitinib and pazopanib, which demonstrated comparable efficacy for PFS and OS [18]. Parametric distributions were fitted to trial-reported Kaplan–Meier curves to estimate PFS/TTP and OS within the model. Based on goodness-of-fit measures (Akaike information criterion, Bayesian information criterion), parametric plots, log cumulative hazard plots, visual inspection, and clinical plausibility of long-term predictions [19], log-normal distribution was used to model PFS/TTP and Weibull distribution to model OS (Supplementary Material A and B in S2 Appendix).

Efficacy inputs for nivolumab and everolimus were derived using patient-level data from CheckMate 025. Efficacy-related statistical significance was assessed using two-sided tests with a significance level of 0.05. To extrapolate clinical outcomes over a patient’s lifetime, parametric distributions [19] were fitted to TTD, TTP, and OS curves. Since Kaplan–Meier curves for nivolumab showed deceleration of hazards after an initial sharp drop [10], none of the single parametric distributions provided a good fit to the observed trial data (Supplementary Material A–C in S3 Appendix). Thus, a dynamic modeling approach was used to project nivolumab by integrating the standard parametric fit from the everolimus arm (Supplementary Material A–C in S4 Appendix) with a Cox proportional hazards regression model. In dynamic modeling, sum of log of hazard ratios obtained from the multivariate Cox proportional hazards regression model is used to shift the reference parametric curve. The proportional hazards assumptions for the treatment arms were assessed by (1) inspecting the cumulative hazards plot (i.e., log-negative-log of survival function vs. log of survival time) to see if it showed non-parallel lines, indicating signs of non-proportionality, and (2) by interacting the coefficient of treatment with log of survival time within the Cox regression analysis to see whether it was significant, indicating non-proportional hazards. It was observed that the proportional hazards assumption was violated; therefore, a piecewise treatment effect (i.e., HR) at 0–3 months and >3 months was used to account for non-proportional hazards.

The impact of MSKCC risk score and objective response on clinical outcomes was analyzed using a Cox proportional hazards regression model. Kaplan–Meier curves for TTD, TTP, and OS were stratified by response status (objective response vs. no objective response) and MSKCC risk score (favorable, intermediate, poor). Visual inspection of the stratified curves indicated that the underlying hazard differed by response status and MSKCC risk score. Parametric survival equations were considered that account for MSKCC risk score and response as baseline predictors. However, as objective response is a time-dependent predictor that can be achieved at a specific time point after treatment initiation and can be lost over time, a dynamic modeling approach was used to avoid bias and account for this time-dependent nature.

A univariate analysis was conducted using patient-level data from CheckMate 025 to evaluate the impact of various patient characteristics, objective response, and nivolumab treatment effect on clinical outcomes. Objective response, MSKCC risk score, and nivolumab treatment effect, before and after a 3-month inflection point, were significant predictors for TTD, TTP, and OS. Hence, a multivariate Cox regression analysis was conducted to quantify the impact of treatment, baseline MSKCC risk score, and time-dependent predictors (e.g., objective response and loss of response) on treatment discontinuation, progression, and death (Supplementary Material A–C in S5 Appendix). The dynamic modeling approach predicted clinical outcomes using a Cox proportional hazards regression model with objective response and treatment effect (0–3 months and >3 months) as time-dependent covariates, and MSKCC risk score as a baseline covariate [20]. The reference group was patients receiving everolimus with no objective response and an MSKCC risk score of poor (Supplementary Material A–C in S4 Appendix).

**Fig 2:**
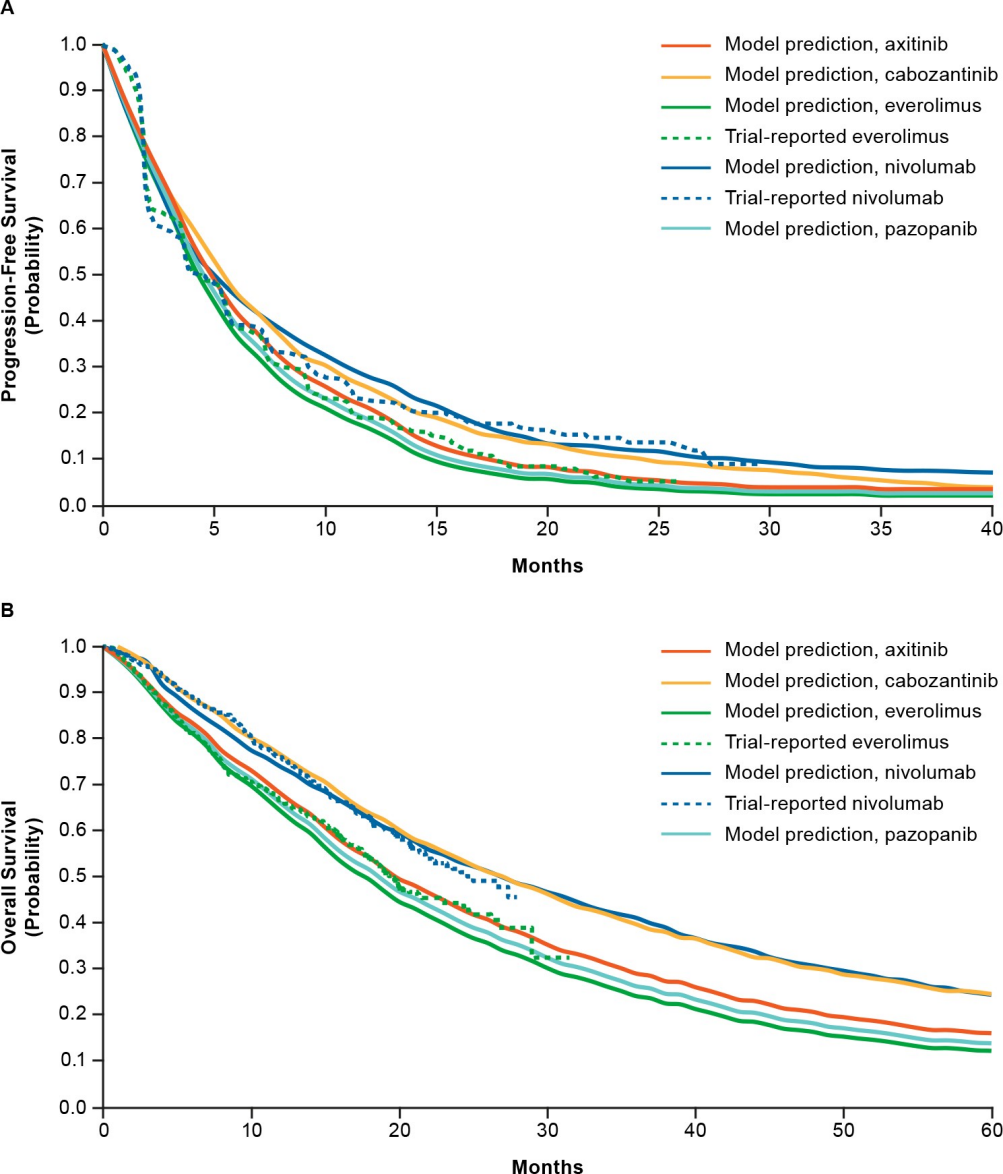
Kaplan–Meier curves showing model predictions for time to progression (A) and overall survival (B) after second-line treatment of advanced RCC (Bristol-Myers Squibb, data on file).

For all other second-line treatments, treatment effect was modeled through application of HRs to the underlying everolimus PFS and OS equations. HRs were derived using indirect treatment comparisons based on published trial results of second-line treatments. In comparison with everolimus, HRs (± standard error) were as follows: PFS, pazopanib (1.64±0.17), axitinib (1.01±0.20), and cabozantinib (0.60±0.07); OS, pazopanib (0.94±0.31), axitinib (0.87±0.10), and cabozantinib (0.67±0.22) (Bristol-Myers Squibb, data on file). Fig 2A and Fig 2B show model-predicted Kaplan–Meier curves for TTP and OS, respectively, for second-line treatments (Bristol-Myers Squibb, data on file).

### Cost inputs

Drug and administration costs were obtained from publicly available sources and derived using recommended dosing (Supplementary Material A and B in S6 Appendix) [7,9,21–26]. AE management costs were calculated for grade 3 or 4 AEs reported in clinical trials [8,10,18,27–29], for which the probability of monthly occurrence was estimated based on reported incidence. The average sunitinib adverse event incidence is based on weighted average of reported incidence for two separate dosing regimens: 75% patients receiving 2 weeks on and 1 week off, and 25% of patients receiving 4 weeks on and 2 weeks off schedule. The majority of the AE management costs considered in the model were taken from Perrin et al 2015 and the Healthcare Cost and Utilization Project-Nationwide Inpatient Sample database (Supplementary Material A and B in S7 Appendix) [30,31]. The distribution of medication received after second-line therapy in CheckMate 025 is shown in S8 Appendix [10]. Average duration of subsequent treatment was 3.5 months, based on clinical trial data [32]. Disease management costs were based on resource use associated with routine management by progression status (S9 Appendix) [31]. All costs were inflated to 2017 US dollars when necessary [26].

### Sensitivity analyses

To explore the uncertainty of model parameters on analysis results, deterministic and probabilistic sensitivity analyses were conducted and results were presented as tornado graphs and cost-effectiveness acceptability curves, respectively. Dirichlet distribution was used for varying MSKCC risk scores and objective response. Multivariate normal distribution and variance-covariance matrices were used for efficacy risk equations; gamma distribution was used for cost inputs and a standard error of 20% of the mean was applied, in accordance with standard guidelines for modeling practices. Probabilistic sensitivity analysis was conducted using 1000 Monte Carlo simulations, each time randomly sampling parameters from individual distributions.

## Results

Table 1 presents cost and clinical outcomes associated with treatment sequences for advanced RCC. The analysis suggests that sequences including nivolumab as second-line treatment were associated with marginally higher life-years (LYs) (4.21 vs. 3.99 years), shorter treatment duration (0.65 vs. 0.72 years), and lower total lifetime costs (first-line sunitinib: $317,056 vs. $335,378; first-line pazopanib: $355,770 vs. $374,093) versus sequences with cabozantinib.

**Table 1:** Health outcomes (years) and costs for sunitinib-and pazopanib-initiating sequences.

First-line treatment	Sunitinib					Pazopanib			
Second-line treatment	Everolimus	Pazopanib	Axitinib	Cabozantinib	Nivolumab	Everolimus	Axitinib	Cabozantinib	Nivolumab
Health outcomes (years)									
First line: Mean time to progression/ treatment discontinuation	0.96	0.96	0.96	0.96	0.96	0.96	0.96	0.96	0.96
Second line									
Mean time to treatment discontinuation	0.38	0.21	0.35	0.72	0.65	0.38	0.35	0.72	0.65
Mean time to progression	0.43	0.21	0.35	0.72	0.81	0.43	0.35	0.72	0.81
Mean survival post second-line treatment	1.45	1.82	1.89	2.31	2.60	1.45	1.89	2.31	2.60
Total life-years	**2.80**	**2.99**	**3.20**	**3.99**	**4.21**	**2.80**	**3.20**	**3.99**	**4.21**
Total quality-adjusted life-years									
Costs									
First-line total	**$151,437**	**$151,437**	**$151,437**	**$151,437**	**$151,437**	**$190,151**	**$190,151**	**$190,151**	**$190,151**
Drug	$127,620	$127,620	$127,620	$127,620	$127,620	$122,915	$122,915	$122,915	$122,915
Adverse event management	$22,970	$22,970	$22,970	$22,970	$22,970	$66,388	$66,388	$66,388	$66,388
Administration	—	—	—	—	—	—	—	—	—
Disease management: Pre-progression	$848	$848	$848	$848	$848	$848	$848	$848	$848
Disease management: Post-progression	—	—	—	—	—	—	—	—	—
Second-line									
Drug	$62,570	$27,069	$54,831	$121,321	$99,870	$62,570	$54,831	$121,321	$99,870
Adverse event management	$9598	$7726	$22,809	$27,605	$3309	$9598	$22,809	$27,605	$3309
Administration	—	—	—	—	$4545	—	—	—	$4545
Disease management: Pre-progression	$277	$187	$308	$592	$283	$277	$308	$592	$283
Disease management: Post-progression	$250	—	—	—	$468	$250	—	—	$468
Second-line total	**$72,696**	**$34,982**	**$77,948**	**$149,518**	**$108,476**	**$72,696**	**$77,948**	**$149,518**	**$108,476**
Subsequent treatment/best supportive care	**$49,671**	**$40,519**	**$37,481**	**$34,423**	**$57,143**	**$49,671**	**$37,481**	**$34,423**	**$57,143**
Total	**$273,804**	**$226,938**	**$266,866**	**$335,378**	**$317,056**	**$312,518**	**$305,580**	**$374,093**	**$355,770**
Cost per life-year	**$97,933**	**$75,814**	**$83,389**	**$84,149**	**$75,268**	**$111,780**	**$95,486**	**$93,863**	**$84,459**

No value is given for post-progression costs in first- and second-line because patients continue to subsequent treatment after progression.

Second-line treatment sequences with everolimus, pazopanib, and axitinib were estimated to accrue similar LYs (around 3 years) and total lifetime costs ($226,938 to $312,518). Primary drivers for total lifetime costs were drug costs (accrued on first- and second-line treatment), which contributed to 65%–75% of total lifetime costs. Based on the analysis, drug costs were highest for cabozantinib ($121,321) followed by nivolumab ($99,870); AE management costs were lowest for nivolumab ($3309) versus other second-line options ($7726–$27,605).

In terms of average cost per LY, nivolumab- and cabozantinib-including sequences were associated with lower costs per LY compared with other second-line treatment options. The analysis suggests that nivolumab-including sequences lead to improved cost/LYs outcomes (first-line sunitinib, $75,268; first-line pazopanib, $84,459) compared with cabozantinib-including sequences (first-line sunitinib, $84,149; first-line pazopanib, $93,863) based on the model-predicted improvements on LY gained and lower per patient costs with nivolumab (Table 2).

**Table 2:** League table for sunitinib- and pazopanib-initiating sequences.

	**Sunitinib-initiating Sequences**		
**Comparator**	**Total costs**	**Total LYs**	**Incremental costs per incremental LY**
Everolimus	$273,804	2.80	Dominated
Pazopanib	$226,938	2.99	Dominant
Axitinib	$266,866	3.20	Extended Dominated
Cabozantinib	$335,378	3.99	Dominated
Nivolumab	$317,056	4.21	$73,927
	**Pazopanib-initiating Sequences**		
**Comparator**	**Total costs**	**Total LYs**	**Incremental costs per incremental LY**
Everolimus	$312,518	2.80	Dominated
Axitinib	$305,580	3.20	Dominant
Cabozantinib	$374,093	3.99	Dominated
Nivolumab	$355,770	4.21	$49,591

LY, life-year.

**Fig 3:**
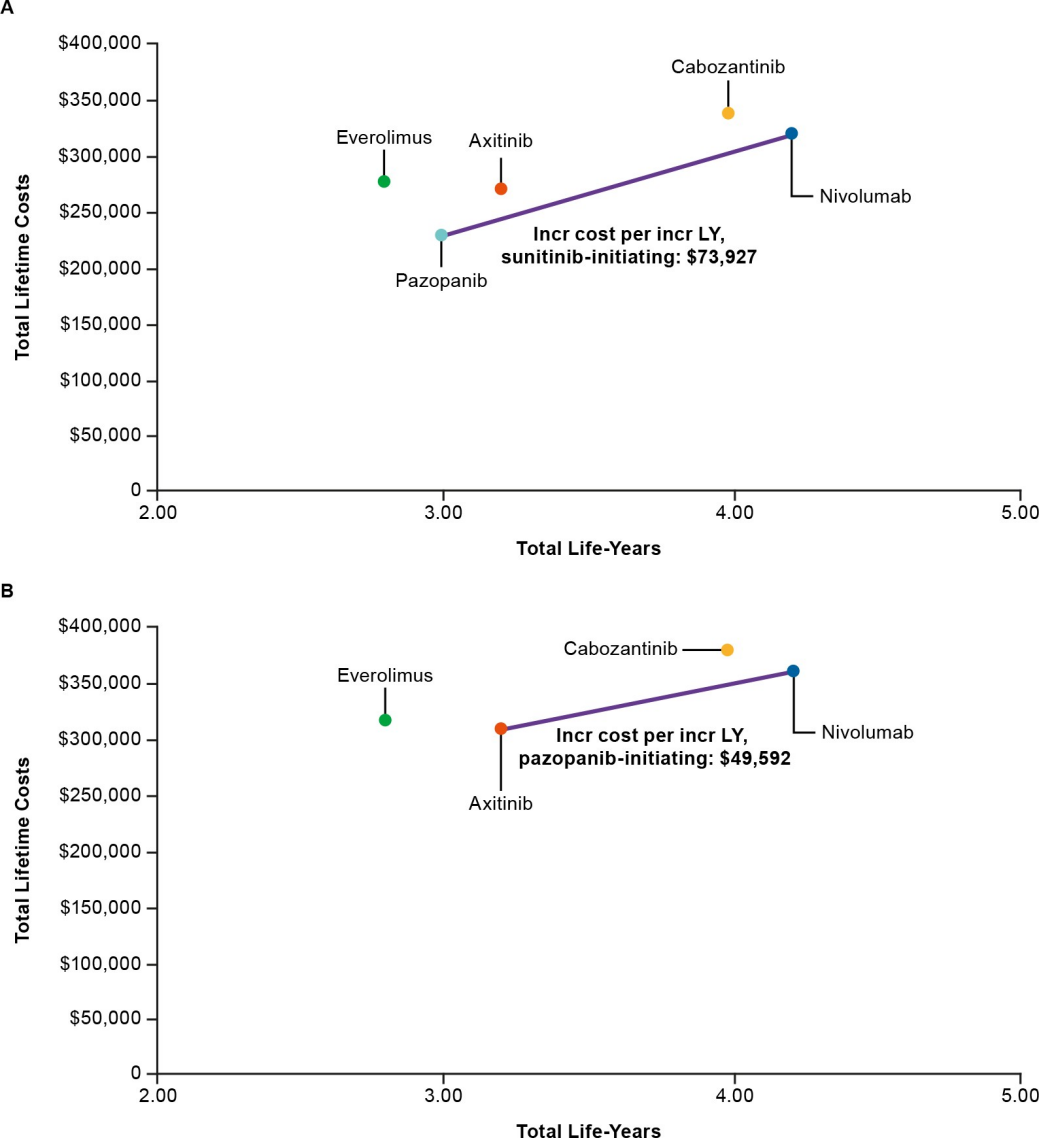
Cost-effectiveness frontiers for sunitinib- (A) and pazopanib-initiating sequences (B).

Incremental cost per incremental LYs gained for second-line sequences including nivolumab versus everolimus was $30,534, versus axitinib was $49,592, and versus pazopanib was $73,927. Fig 3A shows that nivolumab and pazopanib as second-line treatment options are on the cost-effectiveness frontier for sunitinib-initiating sequences with incremental costs per life-year gained of $73,927. For pazopanib-initiating sequences, nivolumab and axitinib are on the cost-effectiveness frontier with incremental costs per life-year gained of $49,592 (Fig 3B).

**Fig 4:**
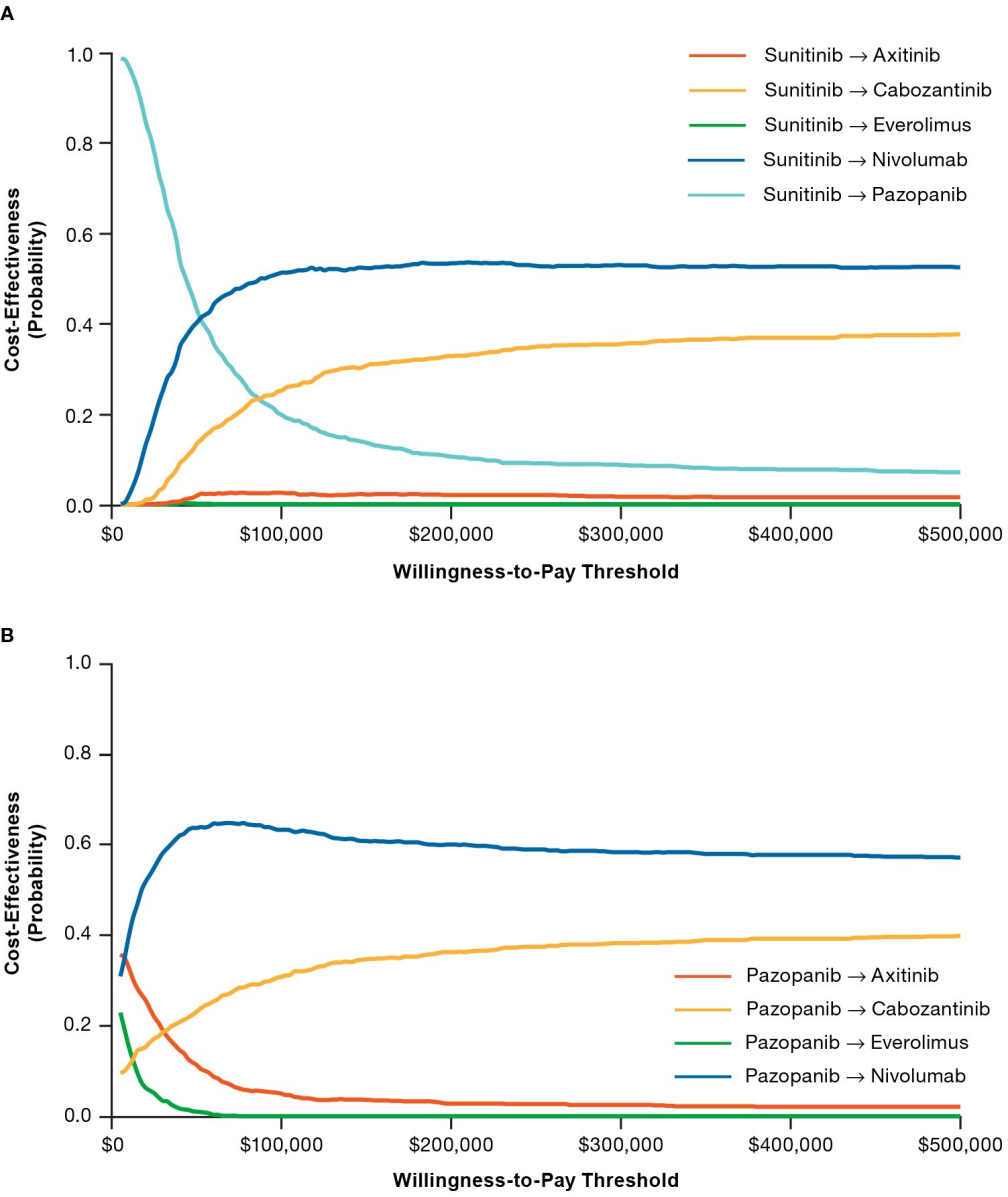
Cost-effectiveness acceptability curves for sunitinib- (A) and pazopanib-initiating sequences (B).

Deterministic sensitivity analysis showed that model results were sensitive to parameters defining the risk equations for TTD, TTP, and OS; HRs versus everolimus in determining treatment effect of various second-line treatment options; and coefficients for MSKCC risk score and response included in the multivariate Cox regression analysis (Figures A–C in S10 Appendix). The cost-effectiveness acceptability curve showed that nivolumab-containing sequences were the most cost-effective strategy at a majority of the willingness-to-pay thresholds (Fig 4).

## Discussion

In this analysis, we assessed the cost and health outcomes associated with treatment sequences used for those patients who continue to progress on first-line treatment for advanced RCC. The treatment sequences included in the analysis are reflective of the current clinical practice, as determined based on NCCN guidelines [13]. Further, key prognostic indicators of health outcomes were assessed, including MSKCC risk score.

Progression and survival are key endpoints generally used to model treatment efficacy [33–35]. However, with the introduction of immuno-oncology drugs, further importance has been given to response evaluation, in terms of rates and durability [15,16]. Thus, this model evaluates the impact of response on progression, treatment discontinuation, and survival. In particular, DICE [17] simulation was used to address heterogeneity in patient populations and the dynamic correlation of response and other disease milestones. This technique allows complex disease modalities and treatment pathways to be modeled in a flexible manner.

Dynamic modeling was used to extrapolate clinical outcomes over a patient’s lifetime; this method uses a flexible Cox proportional hazards model [20], and allows inclusion of both baseline (MSKCC risk score), and more importantly, time-dependent covariates (treatment effect [≤3 and >3 months] and objective response). It was also used to predict clinical outcomes based on changes in surrogate outcomes in real time, such as response achievement/lack of response and loss of response; this method is different from the standard parametric survival analysis (fitting of a single distribution) conducted in previously published analyses [33,34,36]. Although single parametric fits are commonly accepted for targeted agents, they do not accurately capture the predictive role of response on clinical outcomes; this method thus did not provide good fits to the trial data and resulted in lower long-term predictions.

Few patients (7%–8%) were predicted to be alive at the end of the model time horizon (25 years). Survivors were those patients who had achieved and sustained objective response for long durations, resulting in a survival risk similar to that of the general population. Long-term survival impact with checkpoint inhibitors has not yet been studied in a real-world setting, and model results should be validated with data beyond 5 years once available. Despite the longer survival, OS predictions in the model never crossed the US general population mortality estimates [37]. Furthermore, 26-month follow-up data from CheckMate 025 [10] validated the model predictions.

Although a limited number of studies have evaluated treatment sequencing, to the best of our knowledge there are no published reports that have evaluated the economic burden of introducing novel agents within the context of treatment sequences. In this regard, Benedict et al. have estimated an average life-years of 2.90 with sunitinib as first-line treatment option, based on the extrapolation of phase III clinical trial data that compared sunitinib with IFN-alpha [34]. Our analysis estimated that sequences initiating with sunitinib are associated with 2.80–4.20 average life-years. Notably, the higher life-years (3.20–4.20) associated with sunitinib treatment sequences in our study include second-line treatment options such as axitinib, cabozantinib, and nivolumab, which were not available before 2012 [7,9,26].

Other studies that used different methods of extrapolation such as varying parametric equation, spline analyses, and piecewise fits to evaluate cost-effectiveness for second-line treatment options have estimated mean life-years between 1.98–3.44 with nivolumab, 2.10–2.26 with cabozantinib, 1.38–2.09 with axitinib, and 1.73–2.61 with everolimus [38–41]. Despite differences in extrapolation methods, the life-years gained with second-line estimation in our analysis is in agreement with the reported ranges in these published studies. While, our findings are consistent with studies that showed nivolumab is more effective and more cost-effective compared with axitinib and everolimus [38–42], there is less agreement on the cost-effectiveness of nivolumab vs cabozantinib [38,40]. However, the difference in life-years between cabozantinib and nivolumab in these studies is minor with 0.12–0.18 years. This, taken together with cost difference ranging from 3000 to 6000 British pounds indicate that the method of extrapolation for treatment duration and survival likely impacts the cost-effectiveness of nivolumab versus cabozantinib.

This study has several limitations. First, it was assumed that efficacy of second-line treatment was not affected by first-line agent received. This was based on clinical opinion, which suggested that outcomes on second-line treatment are not usually different for patients receiving either sunitinib or pazopanib as first-line treatment. Additionally, the impact of third-line or later treatments on survival was not explicitly modeled. While this is an important consideration, at the time of the study there was no published clinical evidence that could support such analysis. Furthermore, while the impact of third-line treatment on overall survival was not modeled explicitly, survival data used in the assessment (CheckMate-025 and METEOR studies) accounted for the impact of salvage/later-line therapies. Hence, survival estimates used in the model also implicitly incorporate the impact of follow-on therapies.

Another limitation is that HRs based on indirect comparisons (Bristol-Myers Squibb, data on file) were used to estimate the progression and OS for second-line treatments other than nivolumab and everolimus. Simulated trial comparisons/matching-adjusted indirect comparisons were not conducted to account for this variation in baseline characteristics. Further, progression was used as a proxy for treatment duration for sunitinib, pazopanib, axitinib, and cabozantinib, since there were data gaps in published clinical trials for TTD curves [8,18,29]. As CheckMate 025 [10] reported that a subset of patients receive treatment beyond progression (based on RECIST v1.1), using progression as a proxy for treatment duration may not be completely accurate. Finally, the analysis assesses the impact of aggregated experience of grade 3 and 4 AEs. For some AEs, the model assumes that 100% of patients receive inpatient treatment, which may lead to overestimation of AE management costs. Note also that the recently approved combination of lenvatinib and everolimus was not incorporated into the analysis due to insufficient data in advanced RCC. The efficacy and safety of this combination is evaluated in a phase II open-label trial with a small patient population of 153 patients [43]. When phase III trial results are available, future analysis can be expanded to include lenvatinib-based sequences.

## Conclusion

Using a patient-level DICE simulation, the study suggested that, of the treatment sequences evaluated, nivolumab-containing sequences were associated with lowest cost per LY gained. The model suggests that nivolumab-including sequences provide higher LYs gained compared with all other sequences studied. The incremental costs per LY gained for nivolumab is estimated to be well below the commonly used willingness-to-pay threshold in the United States, especially when compared against cabozantinib-including sequences where there is a cost savings.

## Supporting information

S1 Appendix Model simulations. (PDF)

S2 Appendix Standard parametric survival analyses (PFS and OS)–sunitinib and pazopanib for first-line treatment. (PDF)

S3 Appendix Standard parametric survival analyses (TTD, TTP, and OS)–nivolumab and everolimus for second-line treatment. (PDF)

S4 Appendix Standard parametric survival analyses (TTD, TTP, and OS)–everolimus reference arm (MSKCC = poor, objective response = no) for second-line treatment. (PDF)

S5 Appendix Standard parametric survival analyses (TTR and TTLR)–nivolumab and everolimus for second-line treatment and multivariate Cox regression analyses–TTP, TTD and OS for second-line treatment. (PDF)

S6 Appendix Drug and administration costs (per month). (PDF)

S7 Appendix Adverse event costs. (PDF)

S8 Appendix Subsequent treatment costs per month. (PDF)

S9 Appendix Disease management costs per month. (PDF)

S10 Appendix Total life-years and lifetime costs. (PDF)

S11 Appendix Minimal dataset. (XLSX)
