# Supplementary material for: Treatment sequences for advanced renal cell carcinoma: A health economic assessment
Source: PLoS One. 2019 Aug 29;14(8):e0215761. doi: 10.1371/journal.pone.0215761 (PMC6715231; doi:10.1371/journal.pone.0215761)
Supplement: S1 Appendix — (PDF) [file pone.0215761.s001.pdf]

Supplementary Material A. Comparison of CheckMate 025 trial characteristics vs. model simulated characteristics – MSKCC risk score distribution.

	MSKCC Distribution, %	
	**CheckMate 025 (N = 821)[a]**	**Model Simulated (N = 1000)[b]**
MSKCC risk[c]		
Favorable	36	37
Intermediate	49	47
Poor	15	16

[a]Data from Motzer RJ, Escudier B, McDermott DF, et al. Nivolumab versus everolimus in advanced renal-cell carcinoma. N Engl J Med. 2015;373(19):1803-13.
[b]1000 simulations.
[c]Based on interactive voice response system (i.e., randomization stratification level assignment).

MSKCC, Memorial Sloan Kettering Cancer Center.

Supplementary Material B. Comparison of CheckMate 025 trial characteristics vs. model simulated characteristics – Objective response.

	Objective Response, %			
	**CheckMate 025[a]**		**Model Simulated**	
	**Nivolumab**	**Everolimus**	**Nivolumab**	**Everolimus**
MSKCC risk[b]				
Favorable	21	7	22	7
Intermediate	27	5	27	5
Poor	27	0	28	0

[a]Bristol-Myers Squibb, data on file.
[b]Based on interactive voice response system (i.e., randomization stratification level assignment).

MSKCC, Memorial Sloan Kettering Cancer Center.

Supplementary Material C. Model path diagram.

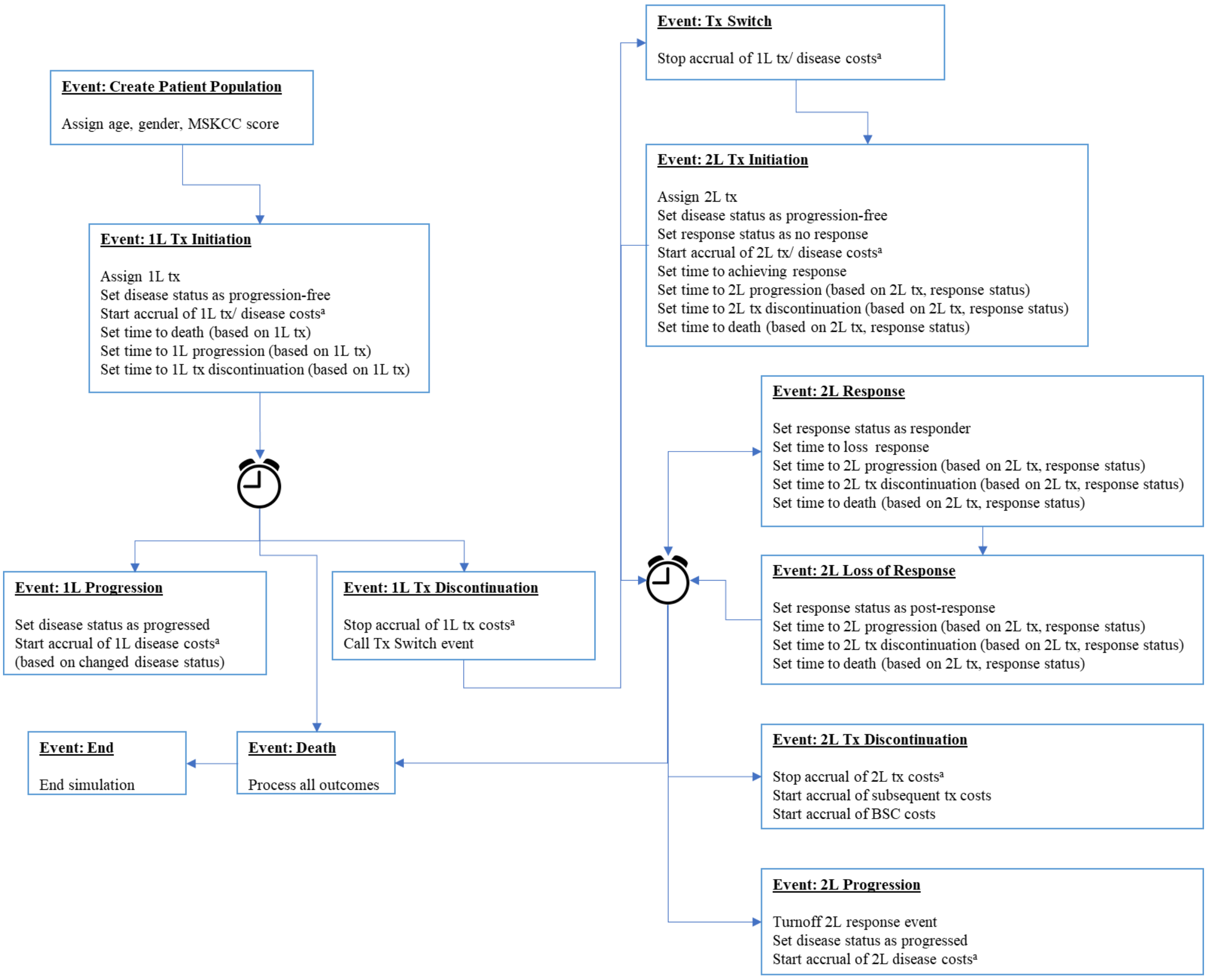

[a]Drug, administration, and adverse event management costs per treatment status; disease management costs (per disease status).

indicates time to event.

1L, first-line; MSKCC, Memorial Sloan Kettering Cancer Center; 2L, second-line; Tx, treatment.
