# Supplementary material for: Treatment sequences for advanced renal cell carcinoma: A health economic assessment
Source: PLoS One. 2019 Aug 29;14(8):e0215761. doi: 10.1371/journal.pone.0215761 (PMC6715231; doi:10.1371/journal.pone.0215761)
Supplement: S2 Appendix — (PDF) [file pone.0215761.s002.pdf]

Supplementary Material A. PFS standard parametric survival analyses: sunitinib and pazopanib for first-line treatment.

Distribution	AIC	BIC	Intercept	Scale/ gamma	Variance: intercept	Covariance: intercept-scale/ gamma	Variance: scale/gamma
Weibull	1377.843	1386.488	2.6694	0.9295	0.002593	0.000188	0.001597
Log-normal	1347.283	1355.928	2.2233	1.1833	0.003298	0.000738	0.002229
Log-logistic	1348.180	1356.825	2.2154	0.6806	0.003063	0.000297	0.00092
Exponential	1378.633	1382.956	2.6792	1.0000	0.0546	–	–
Gompertz	1378.123	1386.768	2.5838	–0.0127	0.006466	0.000485	0.000067

AIC, Akaike's information criterion; BIC, Bayesian information criterion; PFS, progression-free survival.

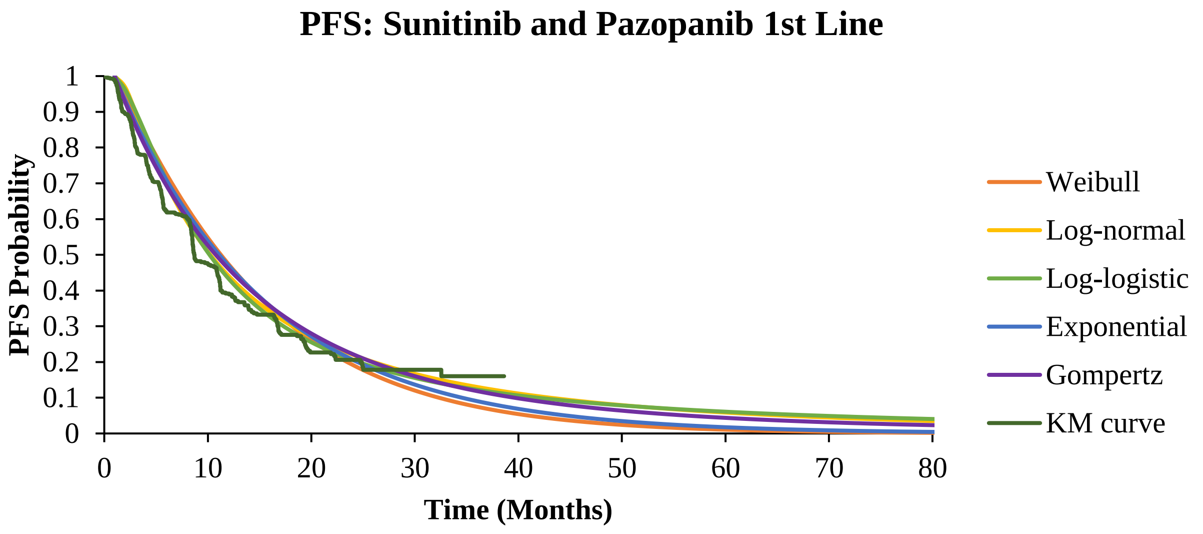

KM, Kaplan–Meier; PFS, progression-free survival.

Supplementary Material B. OS standard parametric survival analyses: sunitinib and pazopanib for first-line treatment.

Distribution	AIC	BIC	Intercept	Scale/ gamma	Variance: intercept	Covariance: intercept-scale/ gamma	Variance: scale/gamma
Weibull	1342.592	1351.237	3.7091	0.8356	0.002139	0.000266	0.00145
Log-normal	1354.039	1362.684	3.3352	1.1836	0.003295	0.000785	0.00228
Log-logistic	1345.076	1353.721	3.3461	0.6645	0.002844	0.00027	0.000904
Exponential	1354.983	1359.306	3.7456	1.0000	0.0547		
Gompertz	1348.897	1357.542	3.9547	0.0105	0.008869	0.000278	0.000013

AIC, Akaike's information criterion; BIC, Bayesian information criterion; OS, overall survival.

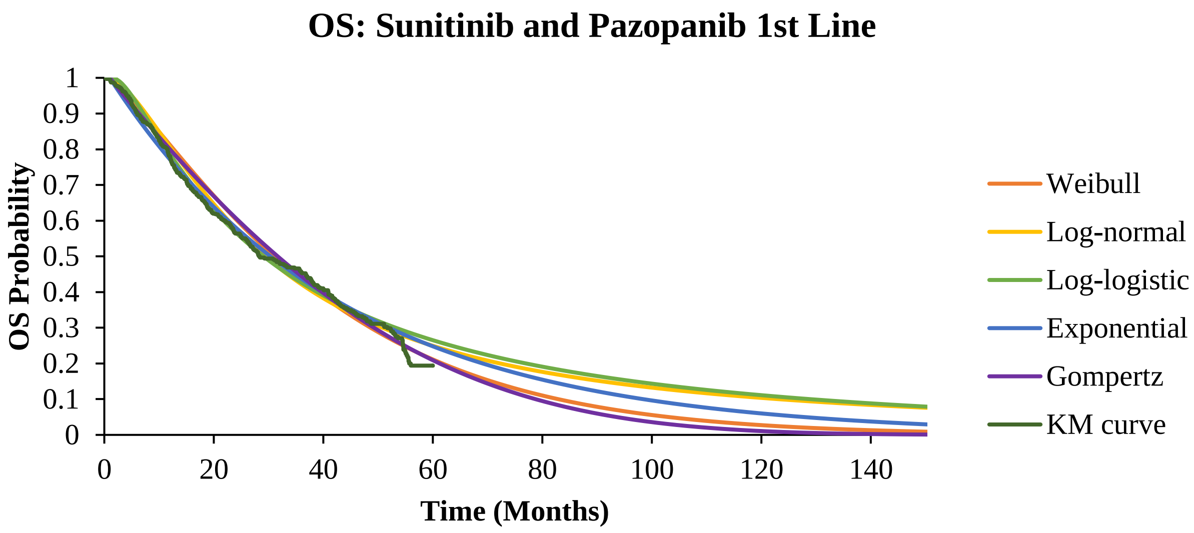

KM, Kaplan–Meier; OS, overall survival.
