# Supplementary material for: Treatment sequences for advanced renal cell carcinoma: A health economic assessment
Source: PLoS One. 2019 Aug 29;14(8):e0215761. doi: 10.1371/journal.pone.0215761 (PMC6715231; doi:10.1371/journal.pone.0215761)
Supplement: S3 Appendix — (PDF) [file pone.0215761.s003.pdf]

Supplementary Material A. TTD standard parametric survival analyses: nivolumab and everolimus for second-line treatment.

Distribution	AIC	BIC	Intercept	Scale/gamma	Variance: intercept	Covariance: intercept-scale/gamma	Variance: scale/ gamma
**Nivolumab**							
Weibull	1242.246	1250.259	2.4241	0.9795	0.002874	–0.000289	0.001887
Log-normal	1204.696	1212.709	1.9347	1.1526	0.003419	0.000264	0.002124
Log-logistic	1210.147	1218.160	1.9260	0.6767	0.003459	0.00008254	0.000922
Exponential	1240.462	1244.469	2.4211	1.0000	0.002949	–	–
Gompertz	1237.299	1245.312	2.2714	–0.0202	0.007021	0.000577	0.000082
**Everolimus**							
Weibull	1200.316	1208.284	1.9977	0.9598	0.002668	–0.000506	0.001496
Log-normal	1133.331	1141.299	1.5101	1.0172	0.002638	0.000069281	0.001458
Log-logistic	1147.357	1155.325	1.4730	0.6032	0.002838	0.000062875	0.000659
Exponential	1199.331	1203.315	1.9846	1.0000	0.00271441	–	–
Gompertz	1196.354	1204.322	1.8545	–0.0216	0.005908	0.000559	0.000098

AIC, Akaike's information criterion; BIC, Bayesian information criterion; TTD, time to treatment discontinuation.

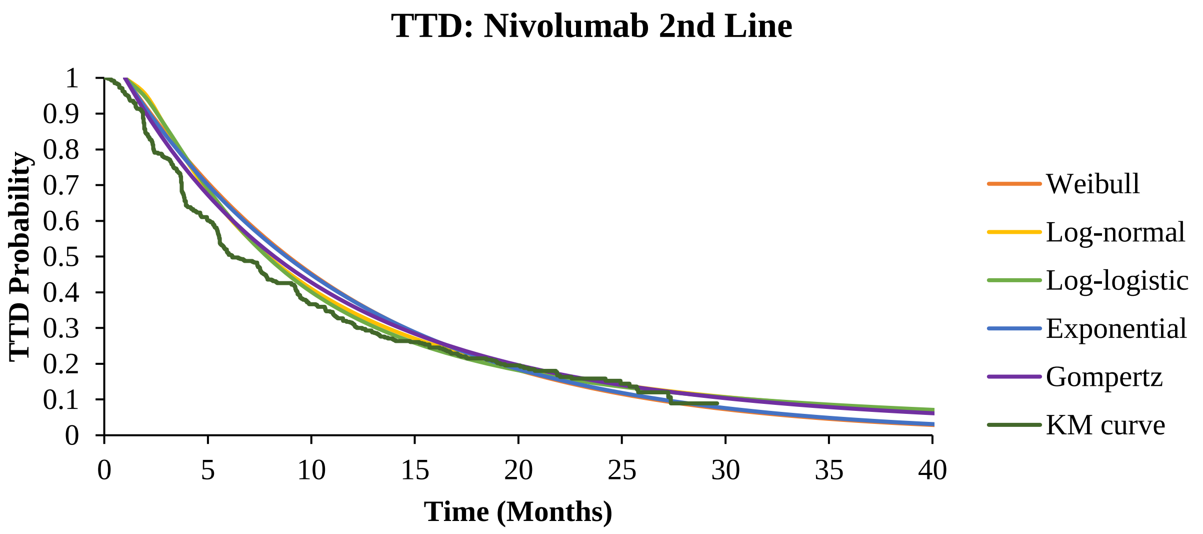

KM, Kaplan–Meier; TTD, time to treatment discontinuation.

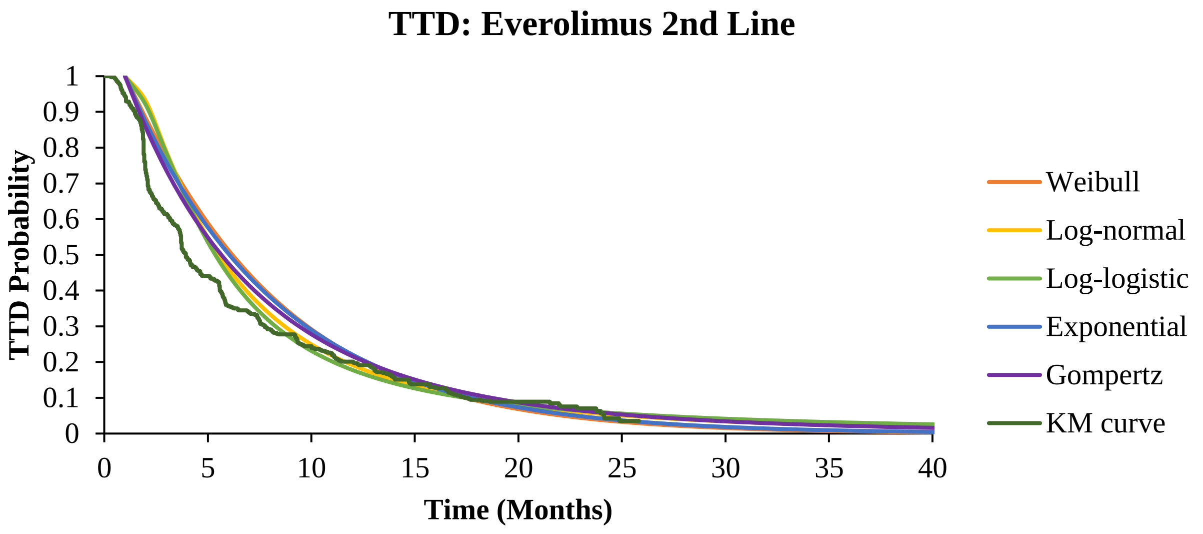

KM, Kaplan–Meier; TTD, time to treatment discontinuation.

Supplementary Material B. TTP standard parametric survival analyses: nivolumab and everolimus for second-line treatment.

Distribution	AIC	BIC	Intercept	Scale/ gamma	Variance: intercept	Covariance: intercept-scale/gamma	Variance: scale/ gamma
**Nivolumab**							
Weibull	1219.053	1227.085	2.1913	1.0953	0.003929	–0.000287	0.002425
Log-normal	1144.055	1152.087	1.6536	1.1902	0.003908	0.000401	0.002511
Log-logistic	1151.124	1159.156	1.5855	0.7069	0.004093	0.000247	0.001081
Exponential	1221.338	1225.354	2.2053	1.0000	0.003249	–	–
Gompertz	1181.883	1189.915	1.7978	–0.0697	0.00656	0.000679	0.000139
**Everolimus**							
Weibull	1048.115	1056.152	2.0561	0.9232	0.002844	–0.000371	0.001611
Log-normal	1005.934	1013.971	1.5763	1.0369	0.003097	0.00024	0.001785
Log-logistic	1014.349	1022.386	1.5576	0.6107	0.003268	0.000125	0.000785
Exponential	1049.363	1053.381	2.0405	1.0000	0.003238	–	–
Gompertz	1050.277	1058.315	1.9748	–0.0114	0.007091	0.000689	0.000123

AIC, Akaike's information criterion; BIC, Bayesian information criterion; TTP, time to progression.

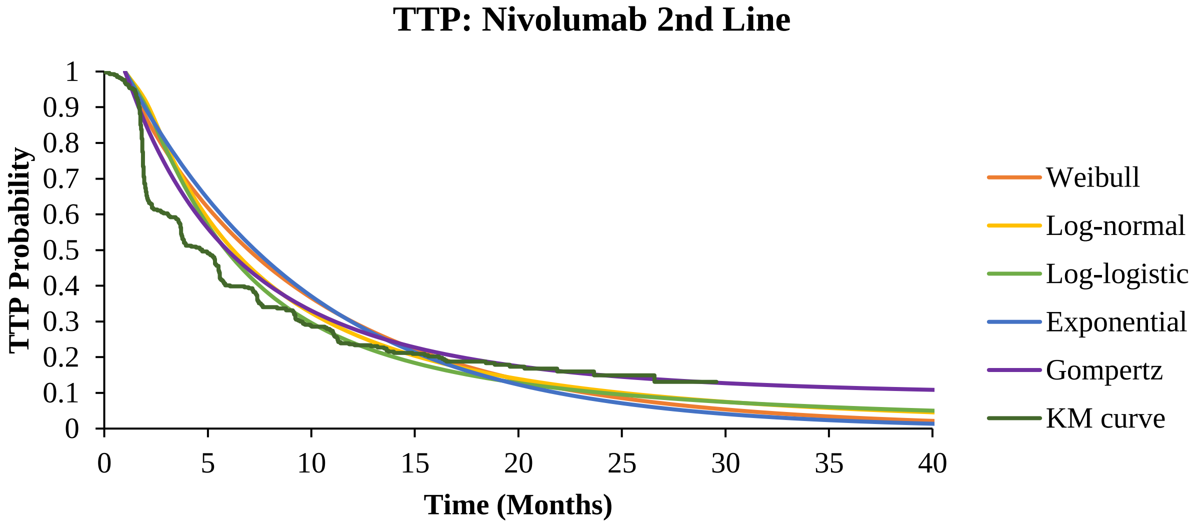

KM, Kaplan–Meier; TTP, time to progression.

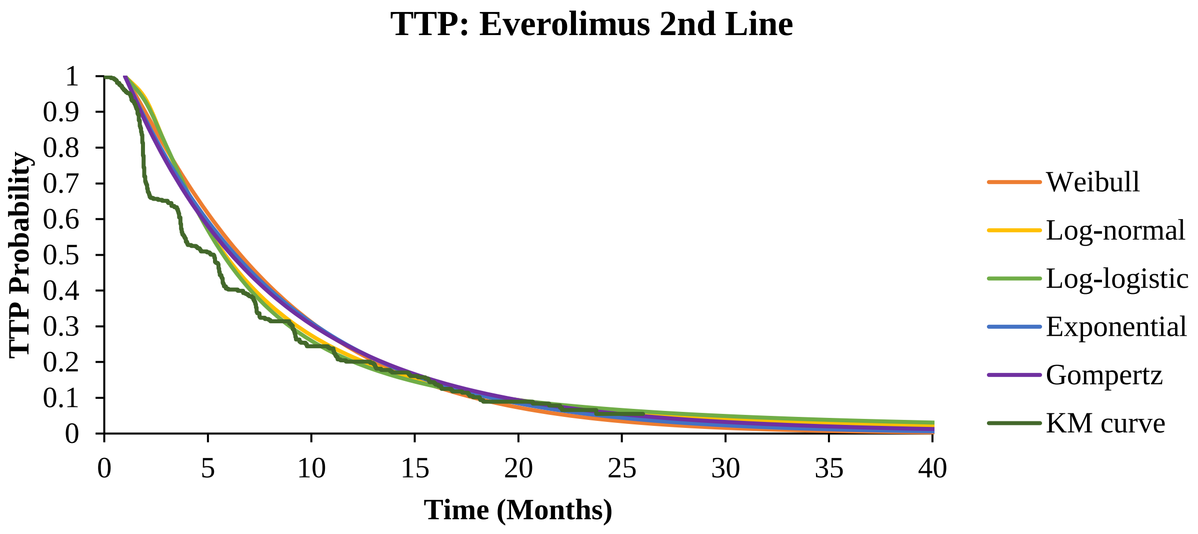

KM, Kaplan–Meier; TTP, time to progression.

Supplementary Material C. OS standard parametric survival analyses: nivolumab and everolimus for second-line treatment.

Distribution	AIC	BIC	Intercept	Scale/gamma	Variance: intercept	Covariance: intercept-scale/ gamma	Variance: scale/gamma
**Nivolumab**							
Weibull	878.662	886.694	3.4885	0.7764	0.004432	0.00177	0.002754
Log-normal	886.486	894.518	3.2776	1.2633	0.006724	0.002981	0.005335
Log-logistic	879.122	887.154	3.2294	0.6744	0.004807	0.001207	0.001959
Exponential	889.408	893.424	3.6376	1.0000	0.005461	–	–
Gompertz	882.781	890.8133	3.9798	0.0315	0.020668	0.001309	0.000113
**Everolimus**							
Weibull	1008.913	1016.951	3.3220	0.9040	0.004392	0.001349	0.003081
Log-normal	1005.134	1013.171	2.9871	1.3419	0.006353	0.002213	0.005032
Log-logistic	1006.472	1014.509	2.9783	0.7603	0.005235	0.000855	0.002017
Exponential	1009.511	1013.529	3.3651	1.0000	0.004651	–	–
Gompertz	1011.068	1019.105	3.4323	0.0067	0.0151	0.001026	0.000101

AIC, Akaike's information criterion; BIC, Bayesian information criterion; OS, overall survival.

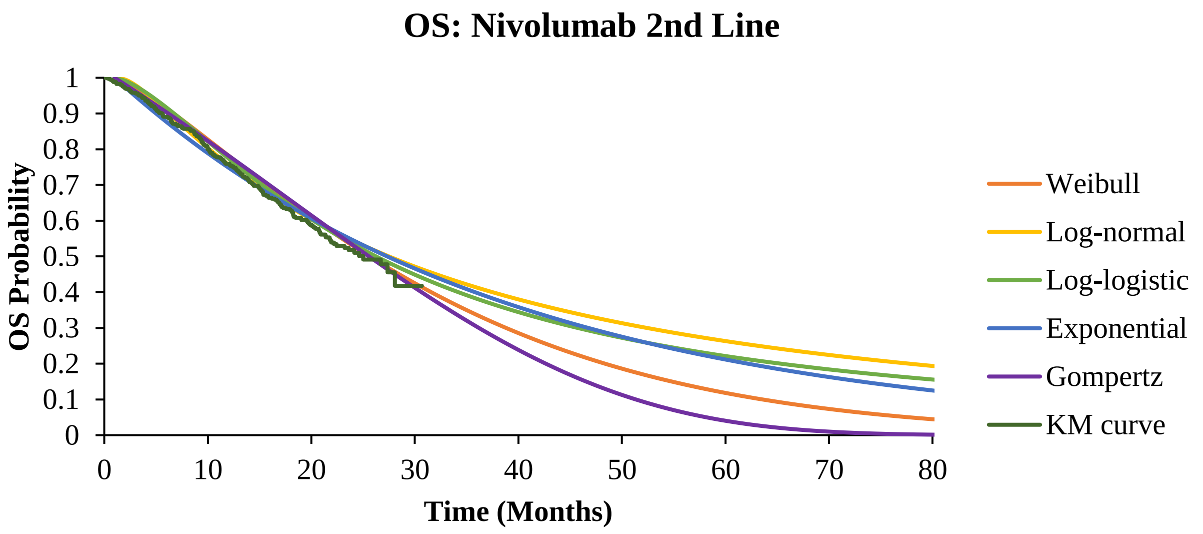

KM, Kaplan–Meier; OS, overall survival.

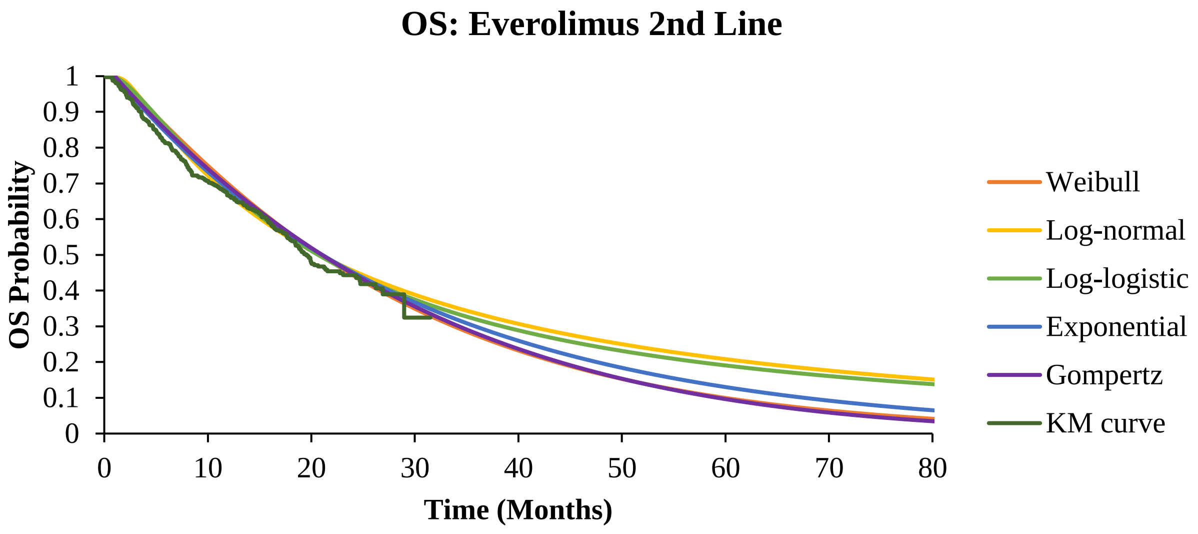

KM, Kaplan–Meier; OS, overall survival.
