# Supplementary material for: Treatment sequences for advanced renal cell carcinoma: A health economic assessment
Source: PLoS One. 2019 Aug 29;14(8):e0215761. doi: 10.1371/journal.pone.0215761 (PMC6715231; doi:10.1371/journal.pone.0215761)
Supplement: S4 Appendix — (PDF) [file pone.0215761.s004.pdf]

Supplementary Material A. TTD standard parametric survival analyses: everolimus reference arm (MSKCC = poor, objective response = no) for second-line treatment.

Distribution	AIC	BIC	Intercept	Scale/gamma	Variance: intercept	Covariance: intercept-scale/ gamma	Variance: scale/gamma
Weibull	167.072	171.193	1.5350	0.9064	0.015983	–0.003666	0.007393
Log-normal	155.574	159.694	1.0781	0.8938	0.013774	–5.80044E–19	0.006887
Log-logistic	156.952	161.073	1.0450	0.5122	0.013845	0.000259	0.003111
Exponential	166.086	168.146	1.4927	1.0000	0.017240	–	–
Gompertz	167.925	172.046	1.4418	–0.0111	0.032868	0.003512	0.000789

AIC, Akaike's information criterion; BIC, Bayesian information criterion; MSKCC, Memorial Sloan Kettering Cancer Center; TTD, time to treatment discontinuation.

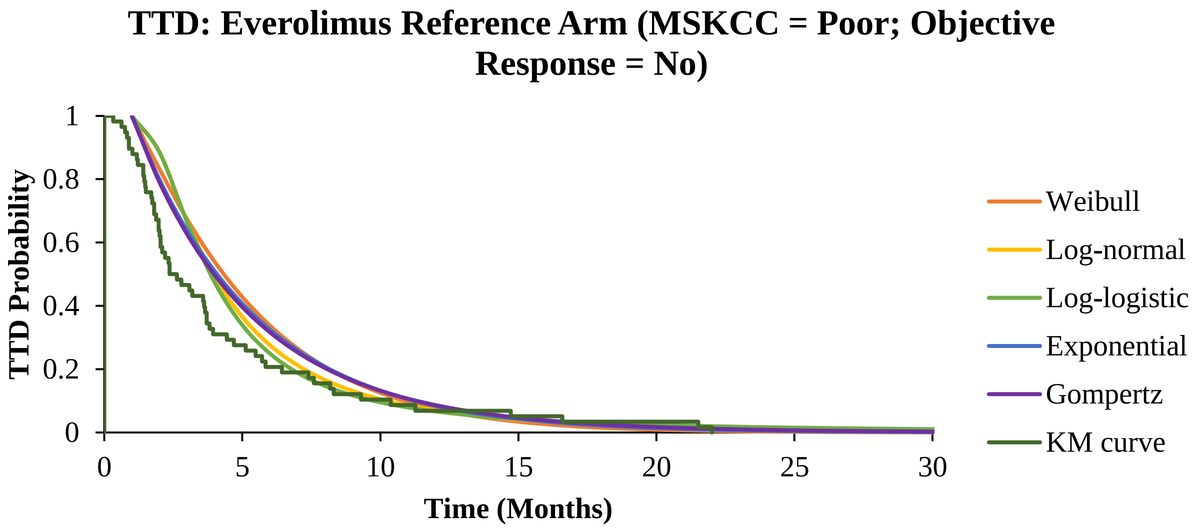

KM, Kaplan–Meier; MSKCC, Memorial Sloan Kettering Cancer Center; TTD, time to treatment discontinuation.

Supplementary Material B. TTP standard parametric survival analyses: everolimus reference arm (MSKCC = poor, objective response = no) for second-line treatment.

Distribution	AIC	BIC	Intercept	Scale/gamma	Variance: intercept	Covariance: intercept-scale/ gamma	Variance: scale/gamma
Weibull	154.330	158.519	1.5437	0.9309	0.019233	-0.003257	0.008987
Log-normal	142.875	147.063	1.0694	0.9263	0.016474	0.000908	0.009014
Log-logistic	143.486	147.675	1.0233	0.5268	0.015993	0.000826	0.004011
Exponential	152.804	154.898	1.5210	1.0000	0.020823	–	–
Gompertz	154.262	158.450	1.4247	-0.0225	0.036983	0.004016	0.000999

AIC, Akaike's information criterion; BIC, Bayesian information criterion; MSKCC, Memorial Sloan Kettering Cancer Center; TTP, time to progression.

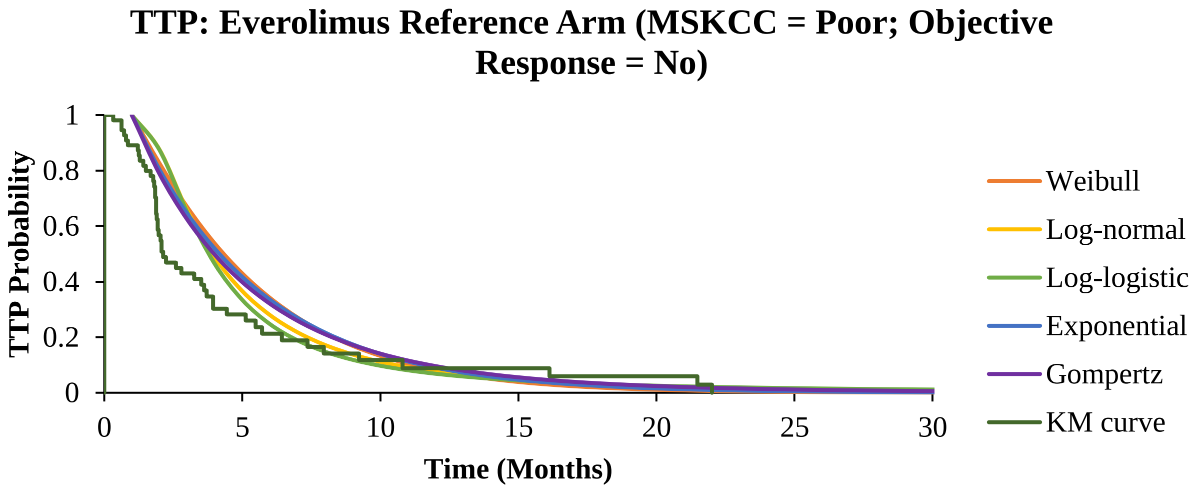

KM, Kaplan–Meier; MSKCC, Memorial Sloan Kettering Cancer Center; TTP, time to progression.

Supplementary Material C. OS standard parametric survival analyses: everolimus reference arm (MSKCC = poor, objective response = no) for second-line treatment.

Distribution	AIC	BIC	Intercept	Scale/gamma	Variance: intercept	Covariance: intercept-scale/gamma	Variance: scale/gamma
Weibull	162.067	166.256	2.5290	0.8245	0.014619	–0.001236	0.00985
Log-normal	157.844	162.033	2.1135	0.9959	0.01827	0.001572	0.011416
Log-logistic	159.403	163.591	2.1173	0.5917	0.018951	0.000376	0.005037
Exponential	162.433	164.528	2.5147	1.0000	0.02128	–	–
Gompertz	164.003	168.192	2.6410	0.0163	0.060254	0.004854	0.000605

AIC, Akaike's information criterion; BIC, Bayesian information criterion; MSKCC, Memorial Sloan Kettering Cancer Center; OS, overall survival.

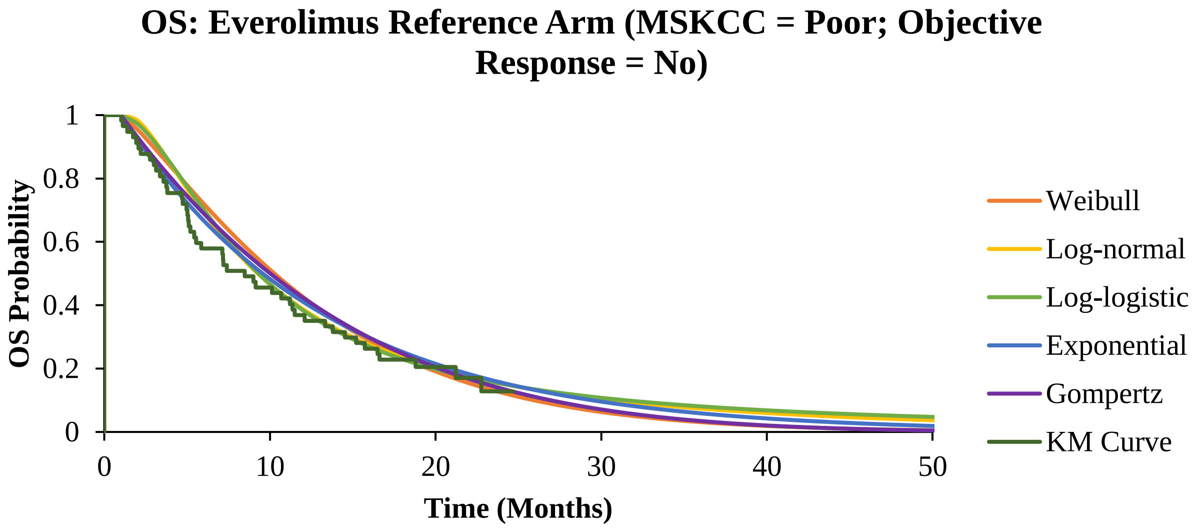

KM, Kaplan–Meier; MSKCC, Memorial Sloan Kettering Cancer Center; OS overall survival.
