# Supplementary material for: Treatment sequences for advanced renal cell carcinoma: A health economic assessment
Source: PLoS One. 2019 Aug 29;14(8):e0215761. doi: 10.1371/journal.pone.0215761 (PMC6715231; doi:10.1371/journal.pone.0215761)
Supplement: S5 Appendix — (PDF) [file pone.0215761.s005.pdf]

Supplementary Material A. TTR standard parametric survival analyses: nivolumab and everolimus for second-line treatment.

Distribution	AIC	BIC	Intercept	Scale/gamma	Variance: intercept	Covariance: intercept-scale/ gamma	Variance: scale/gamma
**Nivolumab**							
Weibull	249.998	255.268	1.5632	0.7646	0.006443	–0.001426	0.002652
Log-normal	208.172	213.441	1.2095	0.6519	0.004126	-7.11641^{-19}	0.002063
Log-logistic	207.328	212.598	1.1369	0.3651	0.003962	0.000153	0.00089
Exponential	261.323	263.957	1.4684	1.0000	0.00970	–	–
Gompertz	262.935	268.205	1.5233	0.0131	0.017731	0.001847	0.000425
**Everolimus**							
Weibull	50.040	52.222	1.6554	0.6111	0.019095	–0.004592	0.009956
Log-normal	47.399	49.581	1.3261	0.6488	0.019135	-1.73814^{-18}	0.009567
Log-logistic	49.538	51.721	1.3062	0.3967	0.022999	0.000278	0.004634
Exponential	55.250	56.341	1.5363	1.0000	0.04545	–	–
Gompertz	52.446	54.628	2.1575	0.1572	0.148635	0.022202	0.004777

AIC, Akaike's information criterion; BIC, Bayesian information criterion; TTR, time to response.

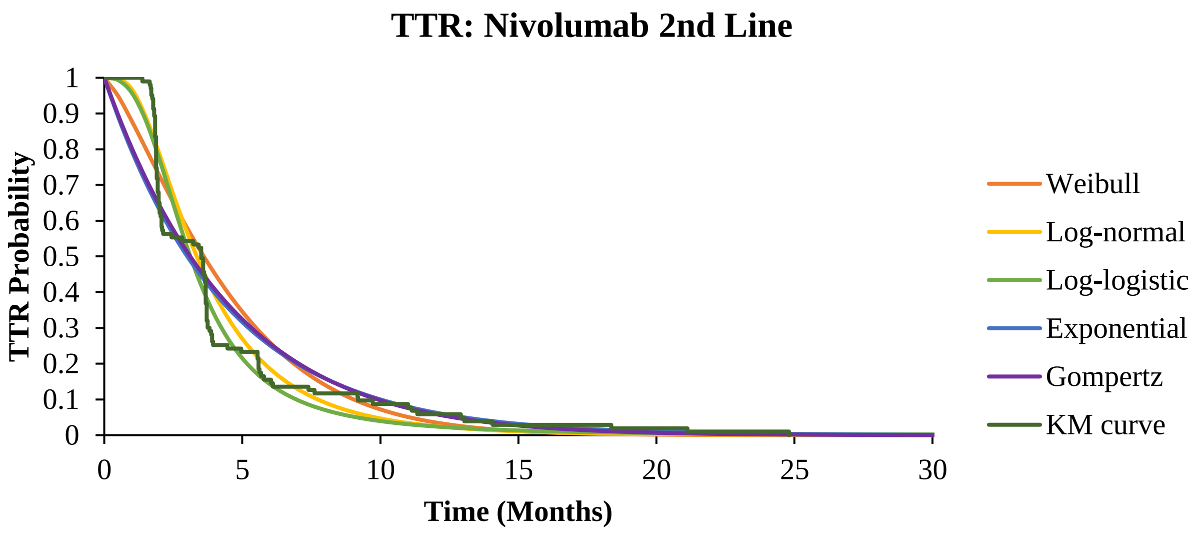

KM, Kaplan–Meier; TTR, time to response.

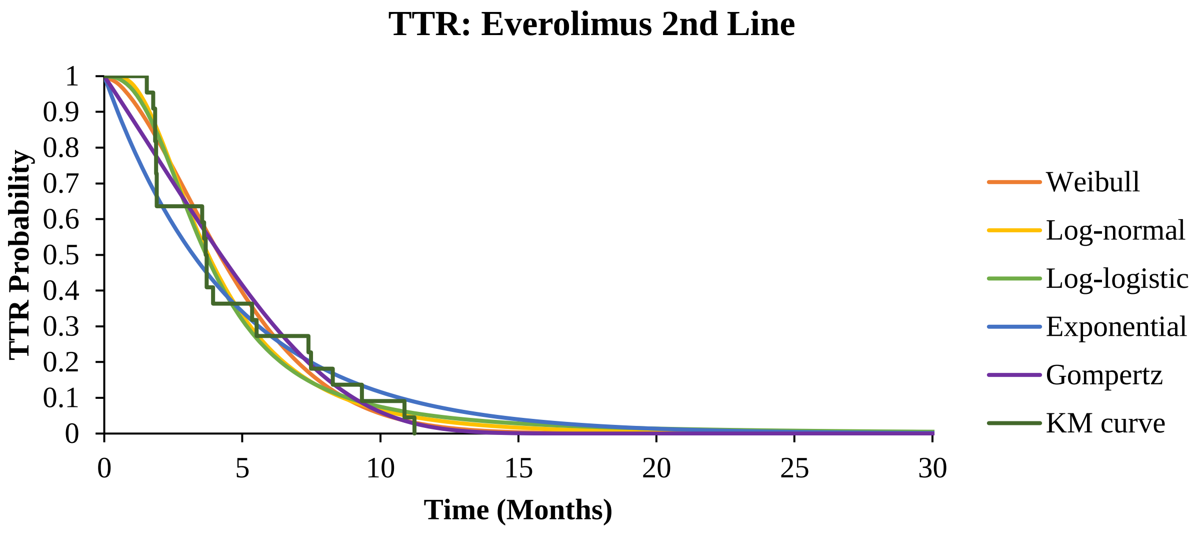

KM, Kaplan–Meier; TTR, time to response.

Supplementary Material B. TTLR standard parametric survival analyses: nivolumab and everolimus for second-line treatment.

**Distribution**	**AIC**	**BIC**	**Intercept**	**Scale/gamma**	**Variance: intercept**	**Covariance: intercept-scale/ gamma**	**Variance: scale/gamma**
**Nivolumab**							
Weibull	308.848	314.118	2.3568	0.8248	0.007109	–0.00154	0.004702
Log-normal	376.746	382.015	1.8022	1.4777	0.021201	$-2.87937E^{-18}$	0.0106
Log-logistic	344.576	349.846	2.0545	0.6582	0.01167	–0.000567	0.003146
Exponential	311.783	314.418	2.3060	1.0000	0.009702	–	–
Gompertz	293.743	299.012	2.9128	0.0702	0.032524	0.002274	0.000227
**Everolimus**							
Weibull	69.015	71.197	2.4066	0.8261	0.033495	–0.007474	0.022556
Log-normal	82.198	84.380	1.8542	1.4309	0.093066	$2.20737E^{-17}$	0.046533
Log-logistic	76.615	78.797	2.0856	0.6720	0.058881	–0.003236	0.014985
Exponential	68.037	69.128	2.3551	1.0000	0.045454	–	–
Gompertz	64.939	67.121	3.0870	0.0804	0.182655	0.013018	0.001235

AIC, Akaike's information criterion; BIC, Bayesian information criterion; TTLR, time to loss of response.

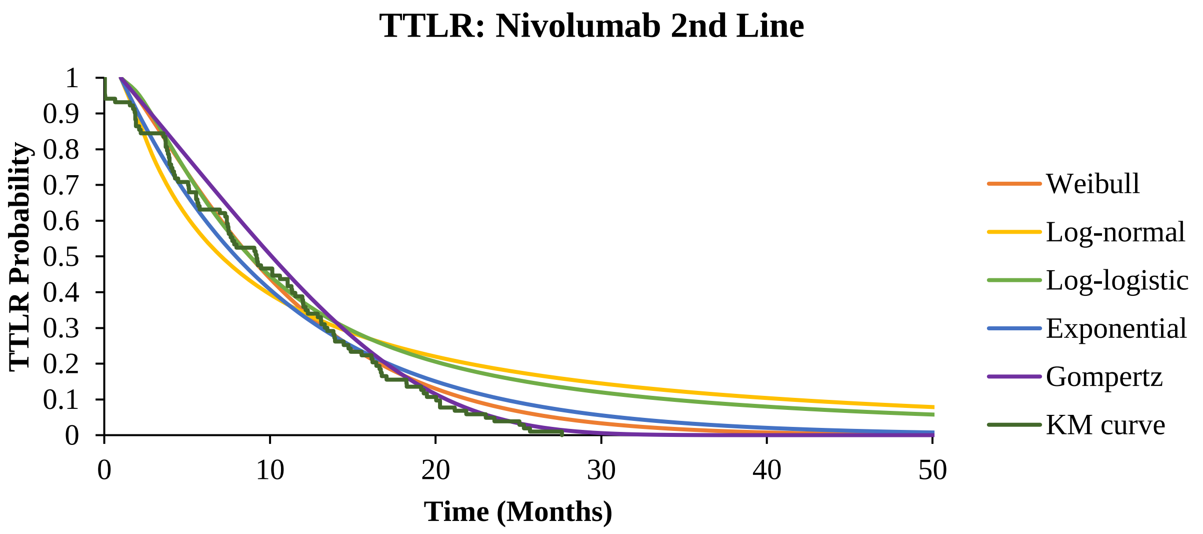

KM, Kaplan–Meier; TTLR, time to loss of response.

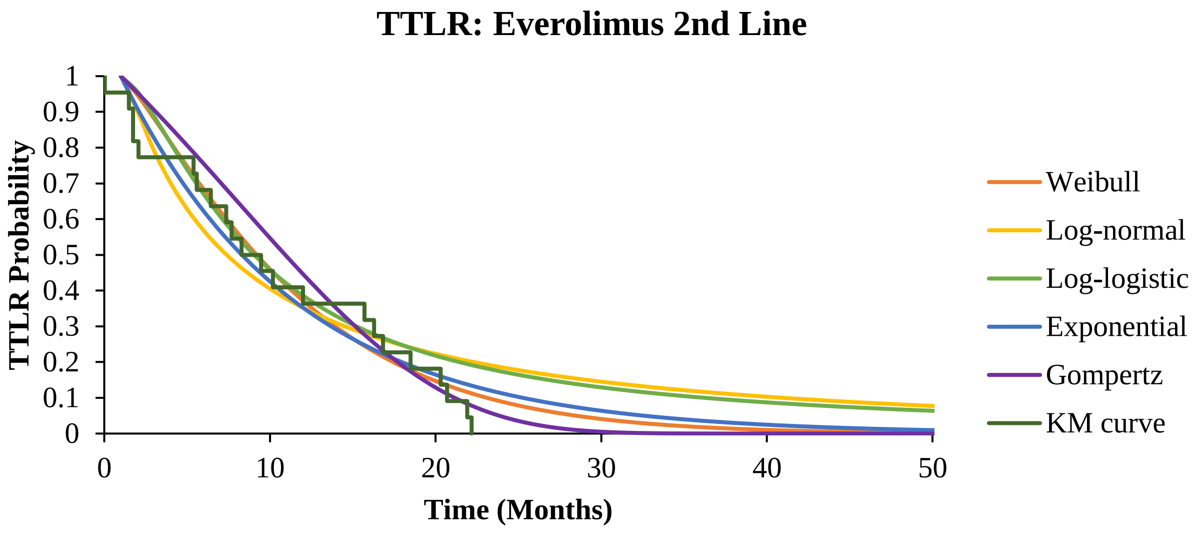

KM, Kaplan–Meier; TTLR, time to loss of response.

Supplementary Material C. Multivariate Cox regression analyses: TTP, TTD and OS for second-line treatment.

		TTD		TTP		OS	
**Variable**	**Level**	**HR estimate (95% CI)**	**p-value**	**HR estimate (95% CI)**	**p-value**	**HR estimate (95% CI)**	**p-value**
Treatment	Everolimus (reference)	1		—[b]	—[b]	—[b]	—[b]
	Nivolumab	0.7270 (0.6240–0.8470)	<0.001	—[b]	—[b]	—[b]	—[b]
Treatment (time-dependent)	Everolimus ≤3 months (reference)	—[a]	—[a]	1		1	
	Nivolumab ≤3 months	—[a]	—[a]	1.1879 (0.9386–1.5033)	0.15	0.5002 (0.2794–0.8955)	0.02
	Everolimus >3 months (reference)	—[a]	—[a]	1		1	
	Nivolumab >3 months	—[a]	—[a]	0.8819 (0.7030–1.1063)	0.28	0.9381 (0.7561–1.1639)	0.56
MSKCC	Poor (reference)	1		1		1	
	Favourable	0.6378 (0.5097–0.7982)	<0.001	0.6323 (0.4955–0.8068)	0.0002	0.2897 (0.2157–0.3892)	<0.0001
	Intermediate	0.7911 (0.6396–0.9786)	0.03	0.7864 (0.6245–0.9904)	0.04	0.6115 (0.4748–0.7876)	<0.001
Response levels (time-dependent)	Pre-response (reference)	1		1		1	
	Objective response	0.1755 (0.1156–0.2663)	<0.001	0.4008 (0.2994–0.5364)	<0.0001	0.0619 (0.0198–0.1934)	<0.001
	Post-objective response	1.3431 (0.9583–1.8823)	0.09	NA	NA	0.4867 (0.2977–0.7956)	0.004

CI, confidence interval; HR, hazard ratio; MSKCC, Memorial Sloan Kettering Cancer Center; NA, not applicable; OS, overall survival; TTD, time to treatment discontinuation; TTP, time to progression.

[a]No value is given for time-dependent treatment effect since treatment discontinuation was associated with overall treatment effect.
[b]No value is given for overall treatment effect since progression and survival were associated with time-dependent treatment effect.
