# Supplementary material for: Treatment sequences for advanced renal cell carcinoma: A health economic assessment
Source: PLoS One. 2019 Aug 29;14(8):e0215761. doi: 10.1371/journal.pone.0215761 (PMC6715231; doi:10.1371/journal.pone.0215761)
Supplement: S6 Appendix — (PDF) [file pone.0215761.s006.pdf]

Supplementary Material A. Drug costs (per month).

Drug	Dose[a]	Frequency[a]	Number of doses per month[a]	Drug acquisition cost[b]	Cost per month
**First line**					
Sunitinib	50 mg	Daily; 4 weeks on, 2 weeks off	30.44	50 mg; 28 tablets = $15,585.35	$11,302
Pazopanib	800 mg	Daily	30.44	200 mg; 120 tablets = $10,718.75	$10,886
**Second line**					
Pazopanib	800 mg	Daily	30.44	200 mg; 120 tablets = $10,718.75	$10,886
Everolimus	10 mg	Daily	30.44	10 mg; 28 tablets = $12,872.74	$14,005
Axitinib	5 mg	Twice daily	30.44	5 mg; 60 tablets = $13,154.07	$13,357
Cabozantinib	60 mg	Daily	30.44	60 mg; 30 tablets = $15,159.38	$15,391
Nivolumab	240 mg	Every 2 weeks	2.17	10 mg/ml; 10 ml solution = $2,545.15<br>10 mg/ml; 4 ml solution = $1,018.06	$13,349
**Subsequent treatments**					
Everolimus	10 mg	Daily	30.44	10 mg; 28 tablets = $12,872.74	$14,005
Axitinib	5 mg	Twice daily	30.44	5 mg; 60 tablets = $13,154.07	$13,357
Pazopanib	800 mg	Daily	30.44	200 mg; 120 tablets = $10,718.75	$10,886
Sunitinib	50 mg	Daily; 4 weeks on, 2 weeks off	30.44	50 mg; 28 tablets = $15,585.35	$11,302
Temsirolimus	25 mg	Weekly	4.35	25 mg/ml; 1 ml solution = $1,676.15	$8,549
Bevacizumab	10 mg/kg	Every 2 weeks	2.17	25 mg/ml; 16 ml solution = $2,967.50	$12,990
Sorafenib	400 mg	Twice daily	30.44	200 mg; 120 tablets = $16,479.43	$16,730
Cabozantinib	60 mg	Daily	30.44	60 mg; 30 tablets = $15,159.38	$15,391

[a]Dosing information obtained from FDA labels.
[b]Drug acquisition cost obtained from Red Book.

Supplementary Material B. Administration costs (per month).

Drug	Route of administration[a]	Number of doses per month[a]	Unit cost[b]	Cost per month
Sunitinib	Oral	30.44	$0	$0
Pazopanib	Oral	30.44	$0	$0
Pazopanib	Oral	30.44	$0	$0
Everolimus	Oral	30.44	$0	$0
Axitinib	Oral	30.44	$0	$0
Cabozantinib	Oral	30.44	$0	$0
Nivolumab	IV infusion: 30 minutes	2.17	$279.45	$607.55

[a]Dosing information obtained from FDA labels.
[b]Drug acquisition cost obtained from Centers for Medicare and Medicaid Services Hospital Outpatient PPS File.
