# Supplementary material for: Treatment sequences for advanced renal cell carcinoma: A health economic assessment
Source: PLoS One. 2019 Aug 29;14(8):e0215761. doi: 10.1371/journal.pone.0215761 (PMC6715231; doi:10.1371/journal.pone.0215761)
Supplement: S7 Appendix — (PDF) [file pone.0215761.s007.pdf]

**Supplementary Material A. Adverse event (grade 3 and 4) cost per episode.**

| Adverse events | Costs per event[a,b] | Adverse events | Costs per event[a,b] |
|---|---|---|---|
| Abdominal pain | $5,487.93 | Hypophosphatemia | $3,135.15 |
| Anaemia | $3,156.20 | Hypotension | $7,976.79 |
| Anorexia | $9,502.04 | Hypothyroidism | $9,454.64 |
| Arthralgia | $55.51 | Increased alkaline phosphatase | $6,540.46 |
| Asthenia | $5,835.53 | Increased ASL | |
| Back pain | $5,540.77 | Increased AST | $7,401.59 |
| Cardiac failure | $10,667.71 | Increased ALT | $7,333.38 |
| Changes in hair colour | | Increased blood cholesterol | $18,312.05 |
| Congestive heart failure | $10,667.71 | Increased blood LDH | $7,333.38 |
| Constipation | $6,131.08 | Increased creatinine | $6,540.46 |
| Cough | | Increase lipase | $6,540.46 |
| Decreased appetite | $6,678.57 | Increased total bilirubin | $11,249.47 |
| Diarrhoea | $6,150.69 | Increased uric acid | $6,822.72 |
| Dizziness | $6,027.65 | Leukopenia | $8,500.12 |
| Dry skin | $6,281.90 | Lymphocytopaenia | $7,517.60 |
| Dysgeusia | | Lymphopenia | $7,517.60 |
| Dyspepsia | $9,861.87 | Malignant neoplasm progr. | $19,953.94 |
| Dysphonia | $7,766.46 | Mucosal inflammation | $8,230.79 |
| Dyspnoea | $5,214.74 | Musculoskeletal pain | $8,699.88 |
| Dyspnoea exertional | $6,366.43 | Nasopharyngitis | $6,165.55 |
| Elevated serum transaminase | $7,333.38 | Nausea | $5,733.53 |
| Epistaxis | $8,617.55 | Neutropenia | $12,429.14 |
| Fatigue | $5,835.53 | Oedema peripheral | $6,880.75 |
| Gastro-oesophageal reflux disease | $7,989.47 | Oropharyngeal pain | $13,317.94 |
| Haemoptysis | $10,199.34 | Pain | $6,504.91 |
| Hand-foot syndrome | $7,038.19 | Pain in a limb | $6,504.91 |
| Headache | $6,779.63 | PPE syndrome | $43.75 |
| Hoarseness | $7,766.46 | Peripheral oedema | $6,817.34 |
| Hypergylcaemia | $4,287.37 | Pleural effusion | $12,840.51 |
| Hypercalcaemia | $10,902.85 | Pneumonia | $9,658.92 |
| Hyperkalaemia | $5,385.72 | Pneumonitis | $5,215.99 |
| Hypermagnesaemia | $6,822.72 | Proteinuria | $4,572.18 |
| Hypertension | $2,926.31 | Pyrexia | $7,055.43 |
| Hypertriglyceridemia | $12,029.45 | Rash | $5,228.28 |
| Hypoalbuminemia | $13,687.46 | Rash maculo-papular | $5,276.91 |
| Hypocalcaemia | $6,960.62 | Stomatitis | $7,959.23 |
| Hypoglycaemia | $7,678.12 | Thrombocytopenia | $10,262.63 |
| Hypokalaemia | $6,723.61 | Urinary tract infection | $7,226.52 |
| Hypomagnesemia | $7,110.37 | Vomiting | $3,757.82 |
| Hyponatremia | $2,926.31 | Weight loss | $7,033.13 |

ALT, alanine aminotransferase; AST, aspartate aminotransferase; LDH, lactate dehydrogenase; PPE palmar-plantar erythrodysesthesia.

[a]Perrin 2015. Inflated to 2017 USD.

[b]Healthcare Utilization Project – National Inpatient Database. Inflated to 2017 USD.

**Supplementary Material B. Adverse event costs per month.**

| Treatment | Cost per month |
|---|---|
| **First-line** | |
| Sunitinib | $2034 |
| Pazopanib | $5880 |
| **Second-line** | |
| Pazopanib | $3107 |
| Everolimus | $2148 |
| Axitinib | $5556 |
| Cabozantinib | $3502 |
| Nivolumab | $442 |
