# Supplementary material for: Treatment sequences for advanced renal cell carcinoma: A health economic assessment
Source: PLoS One. 2019 Aug 29;14(8):e0215761. doi: 10.1371/journal.pone.0215761 (PMC6715231; doi:10.1371/journal.pone.0215761)
Supplement: S8 Appendix — (PDF) [file pone.0215761.s008.pdf]

S8 Appendix. Subsequent treatment costs per month.

Subsequent treatment	Nivolumab arm[a]	Comparator arms[a]
Nivolumab	0%	0%
Everolimus	48%	9%
Axitinib	45%	60%
Pazopanib	17%	26%
Sunitinib	13%	14%
Temsirolimus	5%	5%
Bevacizumab	6%	9%
Sorafenib	12%	15%
Cabozantinib	8%	3%
Total cost per month[b]	$19,282.98	$17,249.78

[a]Subsequent treatment mix from CheckMate 025 trial.
[b]Refer to Supplementary Material A in S6 Appendix.
