# Supplementary material for: Treatment sequences for advanced renal cell carcinoma: A health economic assessment
Source: PLoS One. 2019 Aug 29;14(8):e0215761. doi: 10.1371/journal.pone.0215761 (PMC6715231; doi:10.1371/journal.pone.0215761)
Supplement: S9 Appendix — (PDF) [file pone.0215761.s009.pdf]

S9 Appendix. Disease management costs per month.

Resource name	Number required per month[a]	Unit cost[b]	Cost per month
**Progression-free: $75.07**			
Office visit	0.104	$171.55	$17.84
Community nurse visit	0	$9.33	$0.00
CT scan	0.028	$265.02	$7.42
MRI	0.036	$426.52	$15.35
Blood test, CBC	0.081	$10.66	$0.86
Comprehensive metabolic panel	0.756	$11.60	$8.77
Morphine sulphate, oral	0.019	$60.15	$1.14
Morphine sulphate, IV drug acquisition	0	$18.80	$0.00
Morphine sulphate, IV administration	0.027	$179.77	$4.85
Oxycodone HCl, oral	0.051	$369.02	$18.82
**Progressed: $126.36**			
Office visit	0.188	$171.55	$32.25
Community nurse visit	0	$9.33	$0.00
CT scan	0.044	$265.02	$11.66
MRI	0.047	$426.52	$20.05
Blood test, CBC	0.079	$10.66	$0.84
Comprehensive metabolic panel	0.736	$11.60	$8.54
Morphine sulphate, oral	0.033	$60.15	$1.98
Morphine sulphate, IV drug acquisition	0.001	$18.80	$0.02
Morphine sulphate, IV administration	0.058	$179.77	$10.43
Oxycodone HCl, oral	0.11	$369.02	$40.59

[a]Resource use frequency based on clinical opinion.
[b]Unit costs from RedBook, Centers for Medicare and Medicaid Services – Physician Fee Schedule and Outpatient PPS Files.
