# Supplementary material for: Treatment sequences for advanced renal cell carcinoma: A health economic assessment
Source: PLoS One. 2019 Aug 29;14(8):e0215761. doi: 10.1371/journal.pone.0215761 (PMC6715231; doi:10.1371/journal.pone.0215761)
Supplement: S10 Appendix — (PDF) [file pone.0215761.s010.pdf]

**Figure A. Nivolumab-containing sequences: total life-years.** All data relate to second-line nivolumab unless otherwise stated.

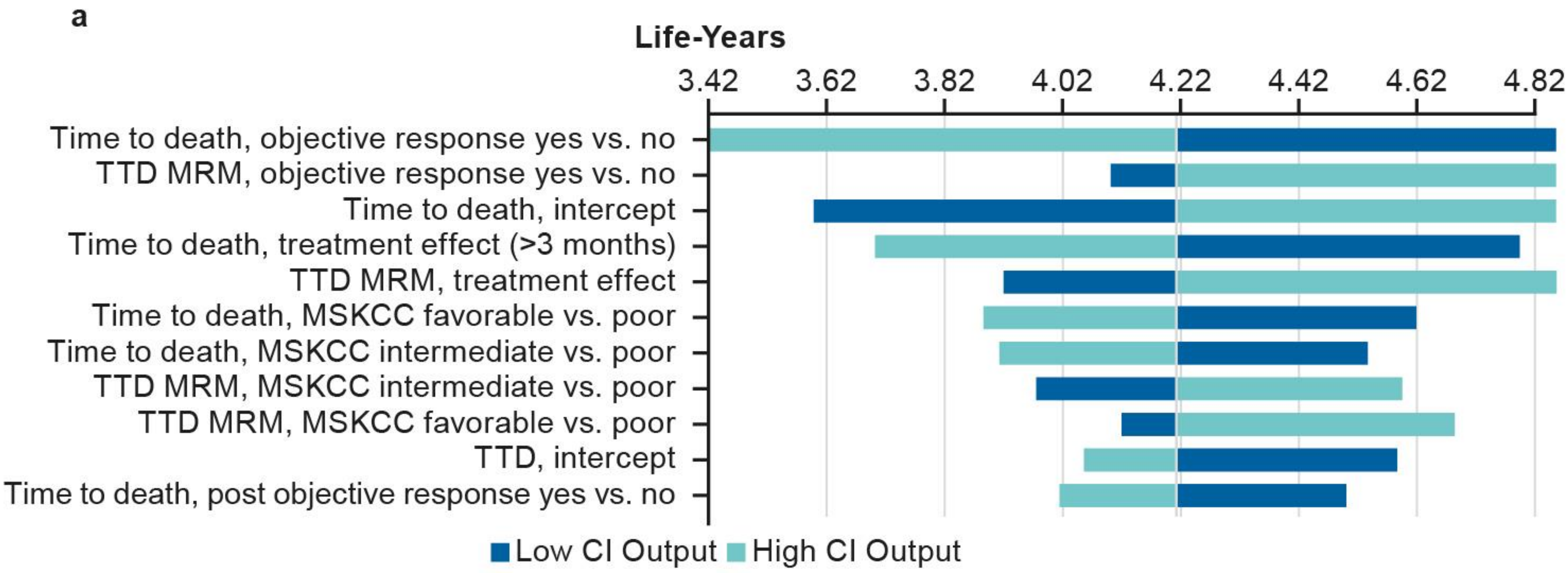


CI, confidence interval; MRM, multivariate regression model; MSKCC, Memorial Sloan Kettering Cancer Center; TTD, time to treatment discontinuation.

**Figure B. First-line sunitinib, second-line nivolumab: total lifetime costs.** All data relate to second-line nivolumab unless otherwise stated.

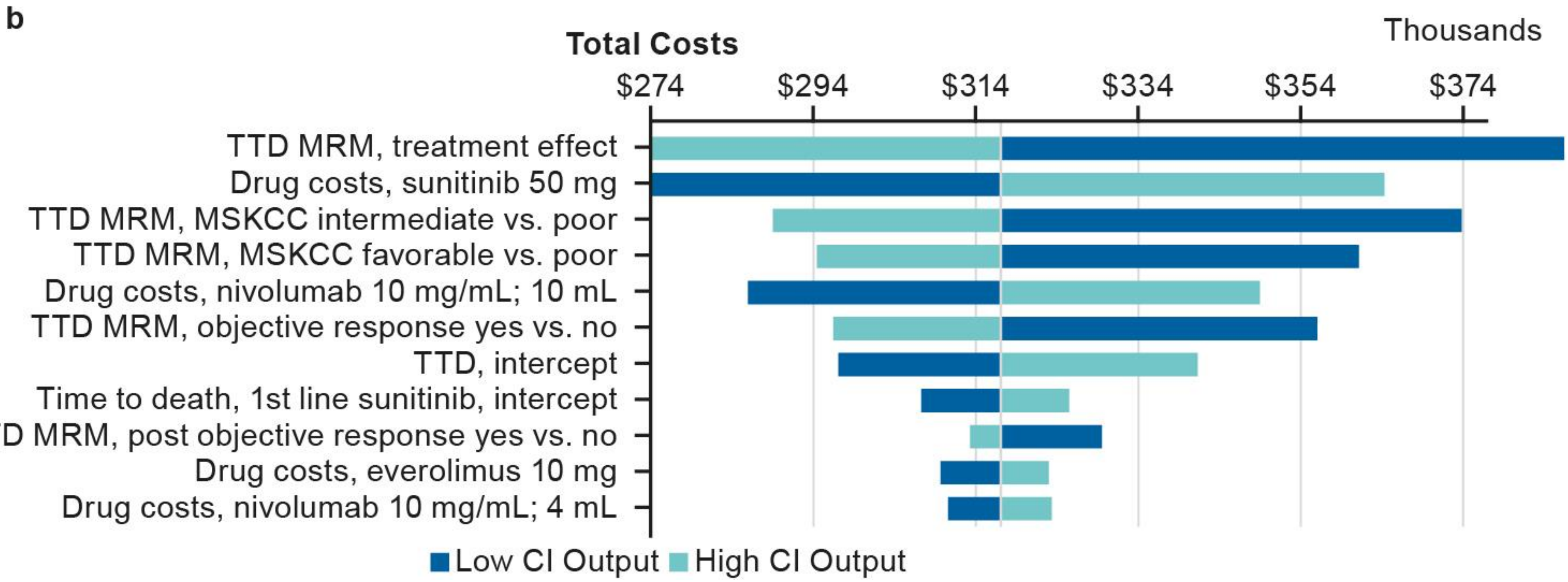


CI, confidence interval; MRM, multivariate regression model; MSKCC, Memorial Sloan Kettering Cancer Center; TTD, time to treatment discontinuation.

**Figure C. First-line pazopanib, second-line nivolumab: total lifetime costs.** All data relate to second-line nivolumab unless otherwise stated.

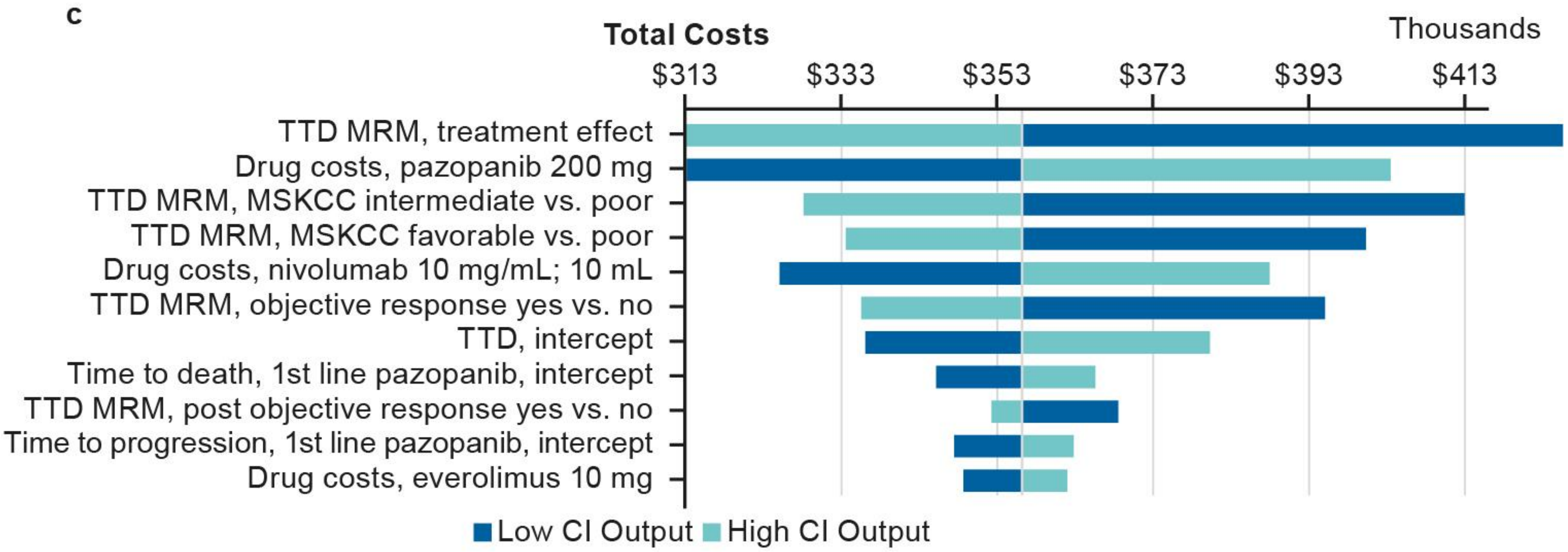


CI, confidence interval; MRM, multivariate regression model; MSKCC, Memorial Sloan Kettering Cancer Center; TTD, time to treatment discontinuation.
